# Mathematical modeling and simulation of thyroid homeostasis: Implications for the Allan-Herndon-Dudley syndrome

**DOI:** 10.3389/fendo.2022.882788

**Published:** 2022-12-08

**Authors:** Tobias M. Wolff, Carina Veil, Johannes W. Dietrich, Matthias A. Müller

**Affiliations:** ^1^ Institute of Automatic Control, Leibniz University Hannover, Hannover, Germany; ^2^ Institute for System Dynamics, University of Stuttgart, Stuttgart, Germany; ^3^ Diabetes, Endocrinology and Metabolism Section, Department of Internal Medicine I, St. Josef Hospital, Ruhr University Bochum, Bochum, Germany; ^4^ Diabetes Centre Bochum-Hattingen, St. Elisabeth-Hospital Blankenstein, Hattingen, Germany; ^5^ Ruhr Center for RareDiseases (CeSER), Ruhr University of Bochum and Witten/Herdecke University, Bochum, Germany

**Keywords:** mathematical modeling, pituitary-thyroid feedback loop, thyroid hormone transport, MCT8 deficiency, Allan-Herndon-Dudley syndrome

## Abstract

**Introduction:**

A mathematical model of the pituitary-thyroid feedback loop is extended to deepen the understanding of the Allan-Herndon-Dudley syndrome (AHDS). The AHDS is characterized by unusual thyroid hormone concentrations and a mutation in the *SLC16A2* gene encoding for the monocarboxylate transporter 8 (MCT8). This mutation leads to a loss of thyroid hormone transport activity. One hypothesis to explain the unusual hormone concentrations of AHDS patients is that due to the loss of thyroid hormone transport activity, thyroxine (*T*_4_) is partially retained in thyroid cells.

**Methods:**

This hypothesis is investigated by extending a mathematical model of the pituitary-thyroid feedback loop to include a model of the net effects of membrane transporters such that the thyroid hormone transport activity can be considered. A nonlinear modeling approach based on the Michaelis-Menten kinetics and its linear approximation are employed to consider the membrane transporters. The unknown parameters are estimated through a constrained parameter optimization.

**Results:**

In dynamic simulations, damaged membrane transporters result in a retention of *T*_4_ in thyroid cells and ultimately in the unusual hormone concentrations of AHDS patients. The Michaelis-Menten modeling approach and its linear approximation lead to similar results.

**Discussion:**

The results support the hypothesis that a partial retention of *T*_4_ in thyroid cells represents one mechanism responsible for the unusual hormone concentrations of AHDS patients. Moreover, our results suggest that the retention of *T*_4_ in thyroid cells could be the main reason for the unusual hormone concentrations of AHDS patients.

## Introduction

The AHDS is a rare and severe disease which was first described in 1944 by Allan, Herndon and Dudley (1). Patients suffer from different symptoms like, e.g., hypotonia, primitive reflexes, scoliosis, muscular hypoplasia and dystonia (2).

A first key observation regarding AHDS patients are low free *T*_4_ (*FT*_4_), slightly elevated thyroid stimulating hormone (*TSH*) and high free triiodothyronine (*FT*_3_) concentrations compared to healthy individuals (2, 3). A second key observation of AHDS patients are mutations in the *SLC16A2* gene encoding for the monocarboxylate transporter 8 (MCT8) (4, 5), which is a specific thyroid hormone transporter (6). A mutation in the related gene often goes along with a complete loss of thyroid hormone transport activity (7).

To elucidate the exact mechanisms that lead to the altered hormone concentrations of this disease, several studies have been made with Mct8 knockout (KO) mice (8–10). These mice miss the MCT8 and are therefore suitable for investigations related to this disease (10). Furthermore, the hormone concentrations of Mct8 KO mice are strongly similar to those of AHDS patients (11).

The thyroids of Mct8 KO mice contain approximately a 3-fold elevation of *T*_4_ compared to wild-type littermates (8). Based on this finding, the hypothesis was made that one mechanism responsible for the unusual hormone concentrations of AHDS patients is a partial retention of *T*_4_ in thyroid cells (12). In this case, more *T*_4_ would be converted into *T*_3_ by thyroidal 5’-deiodinase type I (D1) (12). The result would be that the serum *FT*_4_ concentrations of AHDS patients are lower compared to healthy individuals. Assuming that the *T*_3_ release of the thyroid is not harmed in MCT8-deficiency (which could be explained by further membrane transporters) (8), the serum *FT*_3_ concentrations of AHDS patients would be higher compared to healthy individuals. The feedback signal of *T*_4_ at the pituitary would induce a higher serum concentration of *TSH*.

If there were no additional mechanisms in the genesis of the unusual hormone concentrations than the one described by (12), one would expect that athyroid Mct8 KO mice receiving exogenous thyroid hormone supply do not show the unusual hormone concentrations. In athyroid mice no retention of *T*_4_ in the thyroid can take place and thus the effects of this mechanism should not exist. Investigations with athyroid Mct8 KO mice reveal that it is possible to establish normal *T*_3_ concentrations by exogenous *T*_4_ supply (8). In turn, the *T*_4_ concentrations remain very low (8). This observation indicates that the retention of *T*_4_ in the thyroid gland does contribute to the high *T*_3_ concentrations but not to the low *T*_4_ concentration (12). Therefore, the authors in (12) draw the conclusion that additional mechanisms must contribute to the unusual hormone concentrations. Particularly, they suggest a renal contribution, since *T*_4_ accumulates in the kidney and the activity of D1 inside the kidney is increased for Mct8 KO mice (9).

In this work, we investigate the mechanisms leading to the unusual hormone concentrations of AHDS patients by means of a mathematical model of the pituitary–thyroid feedback loop and dynamic simulations. In detail, we further extend the model originally developed by (13–15) to include membrane transporters exemplarily between the thyroid gland and the periphery. Our results indicate that damaged membrane transporters, i.e., a loss of thyroid hormone transport activity leads to an increased *T*_4_ content in thyroid cells and ultimately to the unusual hormone concentrations that are measured at AHDS patients. These results lead to a partially different conclusion compared to the suggestions given in (12), since they suggest that the entirety of the unusual hormone concentrations of AHDS patients be explained by a retention of *T*_4_ in thyroid cells, without additional renal contribution.

## Methods

First, we introduce the mathematical model of the pituitary-thyroid feedback loop and its extension to membrane transporters. Second, we estimate the unknown parameters using a constrained parameter optimization.

### Extended model

**Figure 1:**
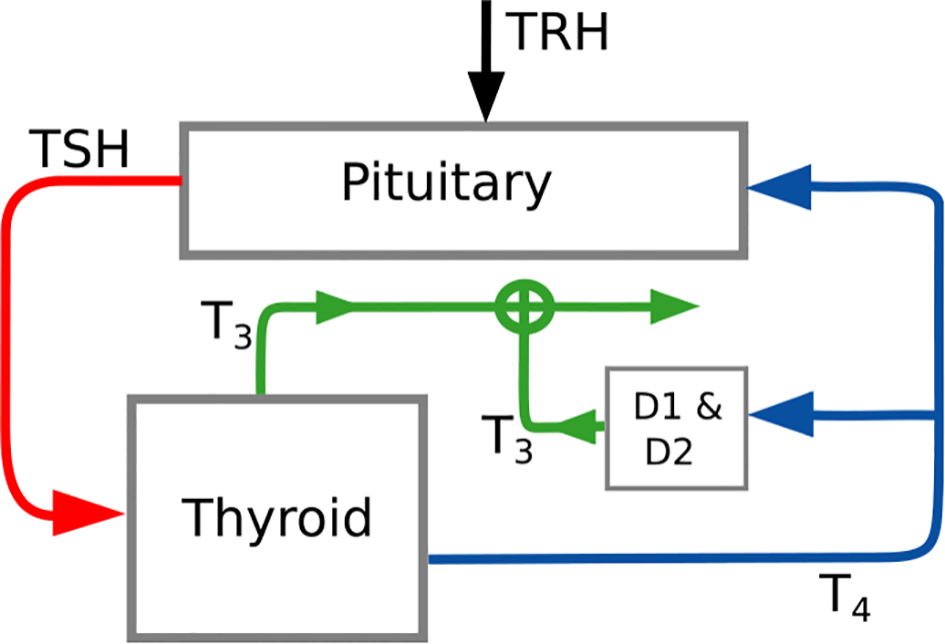
Simplified block diagram of the pituitary thyroid feedback loop. This diagram illustrates the main structure of the applied mathematical model. The detailed model is illustrated in **Figure 2**. The parameter D2 and the variable *TRH* denote the 5’-deiodinase type II and the thyrotropin-releasing hormone, respectively.

**Figure 2:**
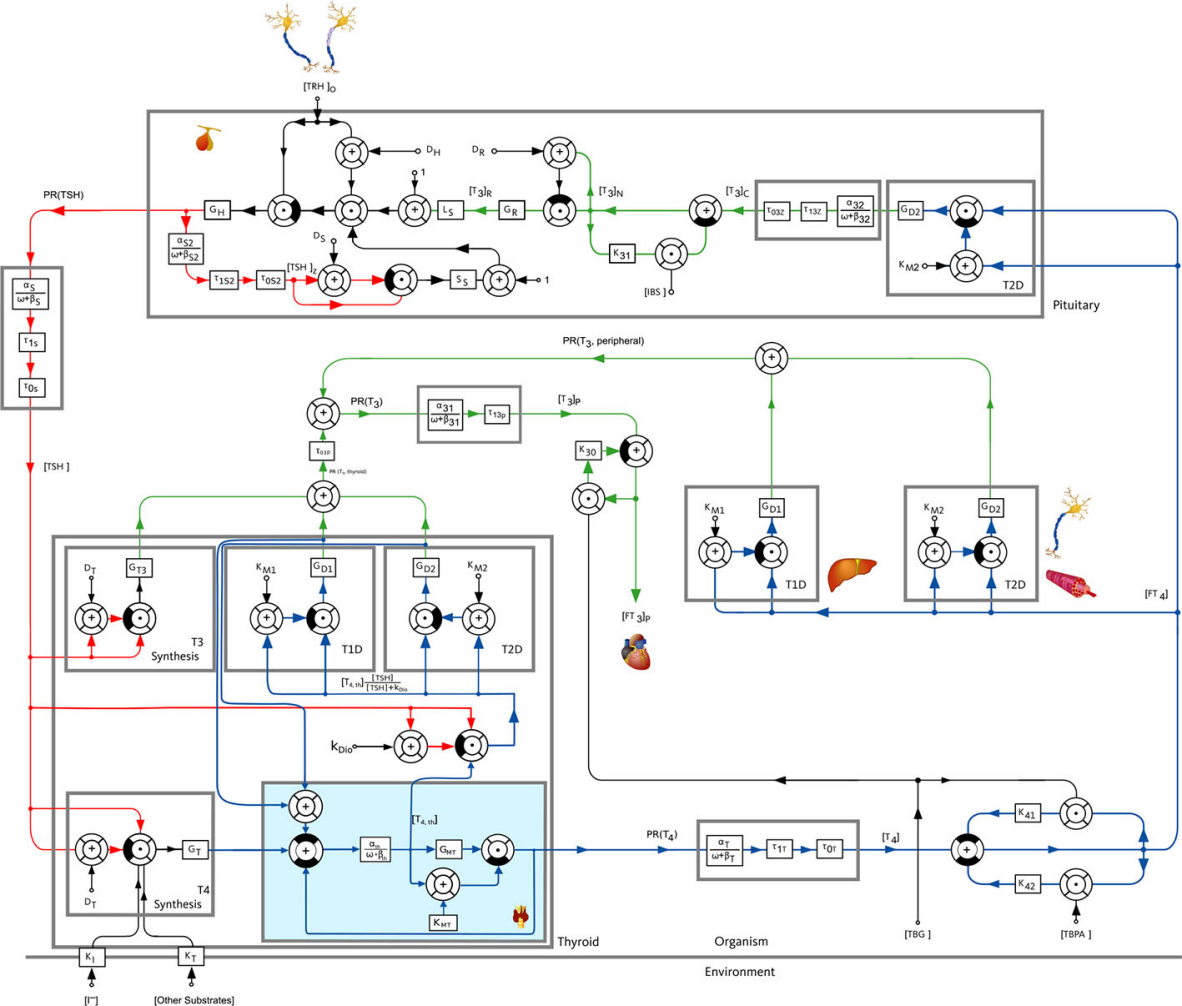
Block diagram of the pituitary-thyroid feedback loop including membrane transporters, extended from (13–15). The extension presented in this paper is shown with a slight blue background color in the block “Thyroid”. The numerical parameter values are mostly taken from (13, 15). The values of G_D1_, G_T3_ and G_MT_ are estimated through a constrained parameter optimization using real hormone measurements from (16).

In general, a mathematical model describes a system by taking into account the available knowledge about the underlying cause-effect relationships. To understand the fundamental principles of the model used throughout this work, a simplified block diagram of it is illustrated in **Figure 1**, whereas a more detailed description is given in **Figure 2**. The basic principle is that *TSH* stimulates the production of *T*_3_ and *T*_4_ at the thyroid gland. By means of D1 and 5’-deiodinase type II (D2), *T*_4_ is converted into *T*_3_ in peripheral organs like the liver and the kidney as well as muscle tissue. The production of *TSH* at the pituitary is inhibited by *T*_4_, whereas the production of *TSH* is stimulated by thyrotropin-releasing hormone (*TRH*). The detailed mathematical model used in this work describes the cause-effect relationships by six nonlinear differential equations visible in **Figure 2**. A detailed state of the art description of the mathematical model as developed by (13–15) is given in Section S1 of the **Supplementary Material**, so that this paper is as self-contained as possible. In the main part, we will emphasize on the extension of the model, conducted within this work.

The investigations of the effects of damaged membrane transporters on the hormone concentrations necessitate a representation of the net effects of the membrane transporters in the mathematical model of the pituitary-thyroid feedback loop from (13–15). For simplicity, we will call the modeled net effects of the membrane transporters “Michaelis-Menten modeling” (or “linear modeling”) of the membrane transporters, even though we do not refer to a specific concentration or state variable but to the net effects.

We incorporate membrane transporters exemplarily between the thyroid gland and the periphery for *T*_4_ (illustrated in **Figure 2** by the slight blue background color) in order to analyze the mentioned hypothesis. Obviously, one could incorporate membrane transporters at a number of different locations in the model, but this would further complicate the model and result in difficulties in the estimation of the additional parameters of the membrane transporters. Moreover, further incorporations of the effects of MCT8 deficiency (or of damaged membrane transporters) are not crucial to pursue the objective of analyzing the hypothesis mentioned in (12) that a retention of *T*_4_ in thyroid cells represents one mechanism responsible for the unusual hormone concentrations of AHDS patients. Furthermore, one could also distinguish between MCT8-mediated and MCT8-independent transport mechanisms. However, the estimation becomes more challenging, if not impossible. Therefore, the here considered membrane transporters can be interpreted as the net effects of all transporters (to which the MCT8 contributes mainly).

The incorporation of the membrane transporters into the already existing model (15) is done by an introduction of a new state, named *T*_4,_ *_th_*. The differential equation of *T*_4,_ *_th_* is defined by


(1) $$\begin{array}{c} \frac{dT_{4,th}}{dt}(t)=\alpha_{th}(G_{T}\frac{TSH(t)}{TSH(t)+D_{T}}-G_{MT}\frac{T_{4,th}(t)}{K_{MT}+T_{4,th}(t)}\\ -G_{D1}\frac{T_{4,th}(t)\frac{TSH(t)}{TSH(t)+k_{Dio}}}{T_{4,th}(t)\frac{TSH(t)}{TSH(t)+k_{Dio}}+K_{M1}}\\ -\;G_{D2}\frac{T_{4,th}(t)\frac{TSH(t)}{TSH(t)+k_{Dio}}}{T_{4,th}(t)\frac{TSH(t)}{TSH(t)+k_{Dio}}+K_{M2}})-\beta_{th}T_{4,th}(t). \end{array}$$

Following the common modeling approach of enzyme/substrate reactions based on the well known Michaelis-Menten kinetics (17), the functionality of the membrane transporters is considered by


(2) $$G_{MT}\frac{T_{4,th}\left(t\right)}{K_{MT}+T_{4,th}\left(t\right)},$$

where *T*_4,_ *_th_* represents the *T*_4_ concentration in thyroid cells. The parameter *G_MT_* stands for the maximal activity of the net effects of all membrane transporters that are involved in the transport of *T*_4_ (for which the MCT8 plays a crucial role). The parameter *K_MT_* is the Michaelis-Menten constant of the membrane transporters. Our model reflects the organ level. Hence, the modeled membrane transporters can be interpreted as the cumulated effect of single membrane transporters on cell level. The complete term thus stands for the part of *T*_4_, which is transported out of the thyroid cells. The first term of (1) describes the production rate of *T*_4,_ *_th_*, with *G_T_* being the secretory capacity of the thyroid gland and *D_T_* representing the damping constant at the thyroid gland (compare Section S1 of the **Supplementary Material** for a more detailed explanation of the model and the meaning of the parameters). The remaining terms represent the thyroidal conversion of *T*_4,_ *_th_* into *T*_3_ by D1 and D2, where the maximal activity of D1/D2 is denoted by *G*_D1_/*G*_D2_ and the dissociation constant of D1/D2 by *K*_M1_/*K*_M2_, respectively^1^. The constant *k_Dio_* is the same in the terms related to D1 and D2 since we assume that the stimulation of the thyroidal D1 and D2 by *TSH* is the same for D1 and D2, compare (15) for a detailed discussion of the *TSH*-*T*_3_ shunt. This conversion rate is considered positively in the differential equation of *T*_3_ *_p_*, equation (S3) of the **Supplementary Material**. Thus, this conversion rate must be considered negatively in the differential equation of *T*_4,_ *_th_*. In (15), these two terms were only present in the differential equation of *T*_3_ *_p_*, because *T*_4,_ *_th_* was not considered as additional state. The constants *α_th_* and *β_th_* are the dilution factor and the clearance exponent for *T*_4,_ *_th_*, respectively. Furthermore, the differential equation of *T*_4_ in plasma changes to


(3) $$\frac{dT_{4}}{dt}\left(t\right)=\alpha_{T}G_{MT}\frac{T_{4,th}\left(t-\tau_{0T}\right)}{K_{MT}+T_{4,th}\left(t-\tau_{0T}\right)}-\beta_{T}T_{4}\left(t\right).$$

The parameters *α_T_* and *β_T_* are again the dilution factor and the clearance rate constant, respectively, for *T*_4_. The dead time *τ*_0_ *_T_* is introduced in order to account for diffusion processes. Compared to (15), the production rate of the peripheral *T*_4_ does no longer correspond to *G_T_ TSH*/(*D_T_* +*TSH*), but to the part of *T*_4,_ *_th_* which is transported out of the thyroid cells.

The numerical values of the majority of the model’s parameters can be taken from (13–15). They have been determined experimentally or derived from known quantities like the half-life of the hormone concentrations. However, the parameters *G_T_*, *G_D_*_1_ and *G_T3_* ^2^ must be re-estimated by fitting them to real measurements of hormone concentrations, because the previous versions of the model did not consider membrane transporters (13–15). In addition, the introduced maximal activity of the membrane transporter *G_MT_* must also be fitted to real hormone measurements. In turn, the Michaelis-Menten constant of the MCT8 for the transport of *T*_4_ was determined experimentally in (6) and its value is applied here for *K_MT_* (although this is a simplification since different mutations will most likely lead to different sensitivities to *T*_4_ and since the net effects of all membrane transporters will also result in a different sensitivity of *T*_4_ compared to the sensitivity of MCT8 alone). This is a meaningful approach, since the Michaelis-Menten constant remains the same for a specific transporter/substrate process (19). To find the numerical parameter values, we neglect the age dependence of the *T*_3_ concentrations of AHDS patients, as documented in (2) and the age dependence of the *T*_4_ content in thyroid cells documented in (20). Even though such a consideration would certainly be advantageous, it is probably not indispensable and would further add complexity to the model.

The factor *α_th_* is defined as the inverse of the volume of distribution of *T*_4_ in the thyroid gland. We choose $\alpha_{th}=250\frac{1}{\text{l}}$ , which corresponds to a volume of distribution of 4 ml. This volume of distribution is based on the assumption that the intracellular parts of the thyroid gland make up one third of the whole volume of the thyroid gland. Furthermore, we choose the clearance exponent for *T*_4_ in the thyroid gland as *β_th_* = 4.4·10^-6^ s^-1^ corresponding to a half-life of *T*_4_ in the thyroid gland of 44 h, a value determined in [20] for rat thyroids. We want to emphasize that the exact numerical values of *α_th_* and *β_th_* do not considerably influence our results. Even if the true values differ to some extent from the ones that we suggest here, our main results (see next section) remain the same, i.e., that the hormone levels of AHDS patients can be explained by damaged membrane transporters. A summary of the entire numerical parameter values can be found in Section S9 in the **Supplementary Material**.

Note that the MCT8 also transports *T*_3_ (21). Membrane transporters for *T*_3_ could be considered in the model by means of an additional state representing the *T*_3_ content in thyroid cells. The mathematical formulation and the results of this consideration are given in Section S6 in the **Supplementary Material**. Problematically, the consideration of membrane transporters for *T*_3_ lead to parameters that are structurally not identifiable using the measurable hormone quantities only. Consequently, our model did not allow to consider membrane transporters for *T*_3_ in a reliable and physiologically meaningful way. Moreover, since the *T*_3_ transport may not be harmed in MCT8 deficiency, compare (8), we do not consider membrane transporters for *T*_3_ in the following. Nevertheless, neglecting membrane transporters for *T*_3_ is a simplification, since it has been explicitly shown that the MCT8 transports *T*_3_ in humans (21).

### Parameter estimation

The parameter estimation of *G_T_*, *G_D_*_1_ *G_T_*_3_ and *G_MT_* is done through a constrained parameter optimization. The idea is to find the optimal configuration of parameters with respect to a cost function by adhering to the system dynamics. We choose the cost function as the normalized quadratic error between real measured hormone concentrations and hormone concentrations computed by the model. Regarding healthy individuals, we use the mean dynamic hormone concentrations documented in (16). In order to quantify the uncertainty related to the parameter estimation, we perform parametric bootstrapping. In other words, we first determine the optimal parameters of the mathematical model using the mean hormone concentrations. The mathematical formulation of this estimation can be found in Section S3 in the **Supplementary Material**. Then, we simulate the mathematical model (using the optimal parameters) 100 times and artificially corrupt the simulated hormone concentrations by noise that follows a normal distribution with *μ* =0 and *σ* =0.1. For each of the 100 datasets, we determine the optimal parameters. Finally, based on this set of optimal parameters, we compute the mean, median, standard deviation and coefficient of variation of all parameters.

For AHDS patients, the estimated (mean) *G_T_*, *G_D_*_1_ and *G_T_*_3_ parameters are held constant and only *G_MT_* is re-estimated for AHDS patients^3^. To this end, we use steady-state hormone concentrations measured in (22–26). Note that, in contrast to the parameter estimation related to healthy individuals, we use steady-state hormone measurements to estimate the *G_MT_* parameter for AHDS patients. To the best of the authors’ knowledge, there are no dynamic hormone measurements of AHDS patients available in the literature that could be applied here. Once again, we perform parametric bootstrapping in order to quantify the uncertainty in the parameters. Here, we use the steady-state hormone concentrations to determine the optimal *G_MT_* parameter for AHDS patients. The exact mathematical formulation of the estimation regarding AHDS patients is given in Section S4 in the **Supplementary Material**. Based on this optimal parameter, we simulate the hormone concentrations of AHDS patients dynamically 100 times and corrupt the simulated *FT*_3_, *FT*_4_ and *TSH* concentrations by some normally distributed noise with *μ* =0 and *σ* =0.1. From here on, we proceed in the same way as for healthy individuals. Note that the estimation of the parameters in both cases requires a normalization of the state variables, which is described in Section S2 in the **Supplementary Material**.

Moreover, for healthy individuals and AHDS patients, we compute the parameters individually. Regarding healthy individuals, we use 27 dynamic hormone measurements documented in (16) and regarding AHDS patients, we use 13 measurements from (22–26). This allows to quantify the variability in the estimated parameters differently, namely by computing the mean, median, standard deviation and the coefficient of variation regarding the individually estimated parameters. Subsequently, we compute a two-sample t-test to analyze whether the mean of the *G_MT_* parameter (describing the maximal activity of the transporters) differs significantly between healthy individuals and AHDS patients for a significance level of 5%. Furthermore, we determine the individual steady-state hormone concentrations of healthy individuals and AHDS patients and analyze whether the mean hormone concentrations differ significantly between healthy individuals and AHDS patients. These results are shown in the **Supplementary Material** in Section S5. Note that one could also perform a parametric bootstrapping for each healthy individual (or AHDS patient) if one is interested in quantifying the *individual* parameter uncertainty. However, this is beyond the scope of this work.

Finally, we perform dynamic simulations to illustrate the course of the hormone concentrations related to healthy individuals and to AHDS patients. We chose arbitrarily a simulation length of 30 days. For the dynamic simulations, we apply the mean parameters (using bootstrapping) determined in both cases. Therefore, the simulation results can be interpreted as hormone concentrations from a generic euthyroid subject and a generic AHDS patient.

When taking a closer look on the expression of (2), one recognizes that a linear approximation of the term is possible if *K_MT_* >> *T*_4,_ *_th_*. By defining the constant *k_l_* = *G_MT_*/*K_MT_*, expression (2) becomes


(4) $$G_{MT}\frac{T_{4,th}}{K_{MT}+T_{4,th}}\approx G_{MT}\frac{T_{4,th}}{K_{MT}}$$

(5) $$=k_{l}T_{4,th}.$$

This means that we can model the functionality of the membrane transporters linearly if the mentioned condition is fulfilled. In this case, equations (1) and (3) must be changed accordingly. The procedure of the parameter estimation remains the same. The only difference is that we have to estimate *k_l_* instead of *G_MT_*. Once again, we perform bootstrapping to quantify the uncertainty of the estimated parameters regarding healthy individuals and AHDS patients. Furthermore, we estimate the parameters individually and quantify the uncertainty based on the individually estimated parameters and show the results in Section S5 of the **Supplementary Material**.

As an additional contribution, we perform a local stability analysis. So far, this was not done for any other version of the here applied mathematical model of the pituitary-thyroid feedback loop (13–15). A stability analysis is interesting for the highly perturbed pituitary-thyroid feedback loop system of AHDS patients. It helps to answer the question whether the feedback loop is stable for damaged membrane transporters. The presentation of the employed method and of the corresponding results are given in the **Supplementary Material** Section S7. This analysis reveals local exponential stability of the equilibrium hormone concentrations.

## Results

First, the results of the parameter estimation and of the dynamic simulations for the Michaelis-Menten modeling of the membrane transporters are presented. Second, the analogous results for the linear modeling of the membrane transporters are shown.

### Michaelis-Menten modeling

The results of the parameter estimation are shown in **Table 1**. This table illustrates the mean, median, standard deviation, and coefficient of variation of all the estimated parameters for healthy individuals and for AHDS patients using bootstrapping. Regarding healthy individuals, one can see that the parameters *G_D_*_1_, *G_T_*, and *G_MT_* show a relatively small coefficient of variation, i.e., a low uncertainty. However, the *G*_T3_ parameter shows a high coefficient of variation, i.e., a high uncertainty. Most importantly, there is great difference in the numerical values between the *G_MT_* parameter for healthy individuals and the one for AHDS patients in addition to the low uncertainty of this parameter. The *G_MT_* parameter related to healthy individuals is approximately 18 times higher compared to the *G_MT_* parameter related to AHDS patients.

**Table 1:** Statistics of the Parameter estimation.

	Healthy Individuals			AHDS Patients	
Parameter	G_D1_ in 10^-8^ mol/s	G_T3_ in 10^-13^ mol/s	G_T_ in 10^-12^ mol/s	G_MT_ in 10^-6^ mol/s	G_MT_ in 10^-6^ mol/s
Mean	2.8053	0.1266	3.2007	2.0196	0.1143
Median	2.7998	0.0237	3.2011	2.0007	0.1125
Standard deviation	0.1205	0.1962	0.0831	0.1706	0.0122
Coefficient of variation	0.0430	1.5502	0.0260	0.0845	0.1067

**Figure 3:**
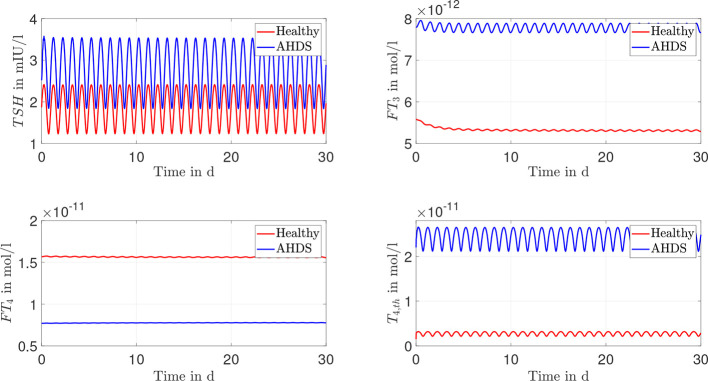
Results of the dynamic simulations for the Michaelis-Menten modeling of the membrane transporters. Most of the numerical parameter values are based on the suggestions of (13, 15). The remaining unknown parameters of the model are estimated through a constrained parameter optimization and shown in **Table 1**.

Once the unknown parameter values are determined, dynamic simulations can be performed using the mean parameter estimates as documented in **Table 1**. The course of the hormone concentrations of *TSH*, *FT*_4_, *FT*_3_ and *T*_4,_ *_th_* are given in **Figure 3**, where a sinusoidal *TRH* input was used (as an approximation of the real pulsatile *TRH* course). Regarding the hormone concentrations, one can see that the *FT*_3_ and the *TSH* concentrations are approximately 1.4 times higher for AHDS patients compared to healthy individuals. In turn, the *FT*_4_ concentration is 2.1 times higher for healthy individuals compared to AHDS patients. Interestingly, the *T*_4,_ *_th_* concentration is approximately 18 times higher for AHDS patients compared to healthy individuals. Note the advantage of applying a mathematical model: we are able to make conclusions about the *T*_4_ content in thyroid cells which cannot be measured in AHDS patients.

### Linear modeling

We now focus on the results of the linear approximation of the membrane transporters. As can be seen from **Figure 3** and Section S9 of the **Supplementary Material**, the assumption *K_MT_* = 4.7·10^-6^ >> *T*_4,_ *_th_* ≈ 2.5·10^-12^ is fulfilled. Therefore, the same analysis as in the previous subsection is performed with the linear approximation of the membrane transporters. The results of the parameter estimation are shown in **Table 2**.

**Table 2:** Statistics of the Parameter estimation.

	Healthy Individuals			AHDS Patients	
Parameter	G_D1_ in 10^-8^ mol/s	G_T3_ in 10^-13^ mol/s	G_T_ in 10^-12^ mol/s	*K_l_* in 1/s	*K_l_* in 1/s
Mean	2.8112	0.1450	3.1945	0.4327	0.0239
Median	2.7870	0.0265	3.2000	0.4275	0.0239
Standard deviation	0.1274	0.1943	0.0882	0.0402	0.0021
Coefficient of variation	0.0453	1.3398	0.0276	0.0928	0.0862

The mean values of the *G_D1_ G_T3_*, and *G_T_* parameters of the linear modeling of the membrane transporters are similar to the ones of the Michaelis-Menten modeling. The mean value *k_l_* = 0.4327 for healthy individuals (*k_l_* = 0.0239 for AHDS patients) corresponds approximately to *G_MT_*/*K_MT_* = 0.4297 (*G_MT_*/*K_MT_* = 0.0243 for AHDS patients), compare **Table 1** and Section S9 of the **Supplementary Material**. Once again, the value of *k_l_* is approximately 18 times higher for healthy individuals compared to AHDS patients. The uncertainty related to the parameters is similar compared to the Michaelis-Menten modeling of the membrane transporters.

**Figure 4:**
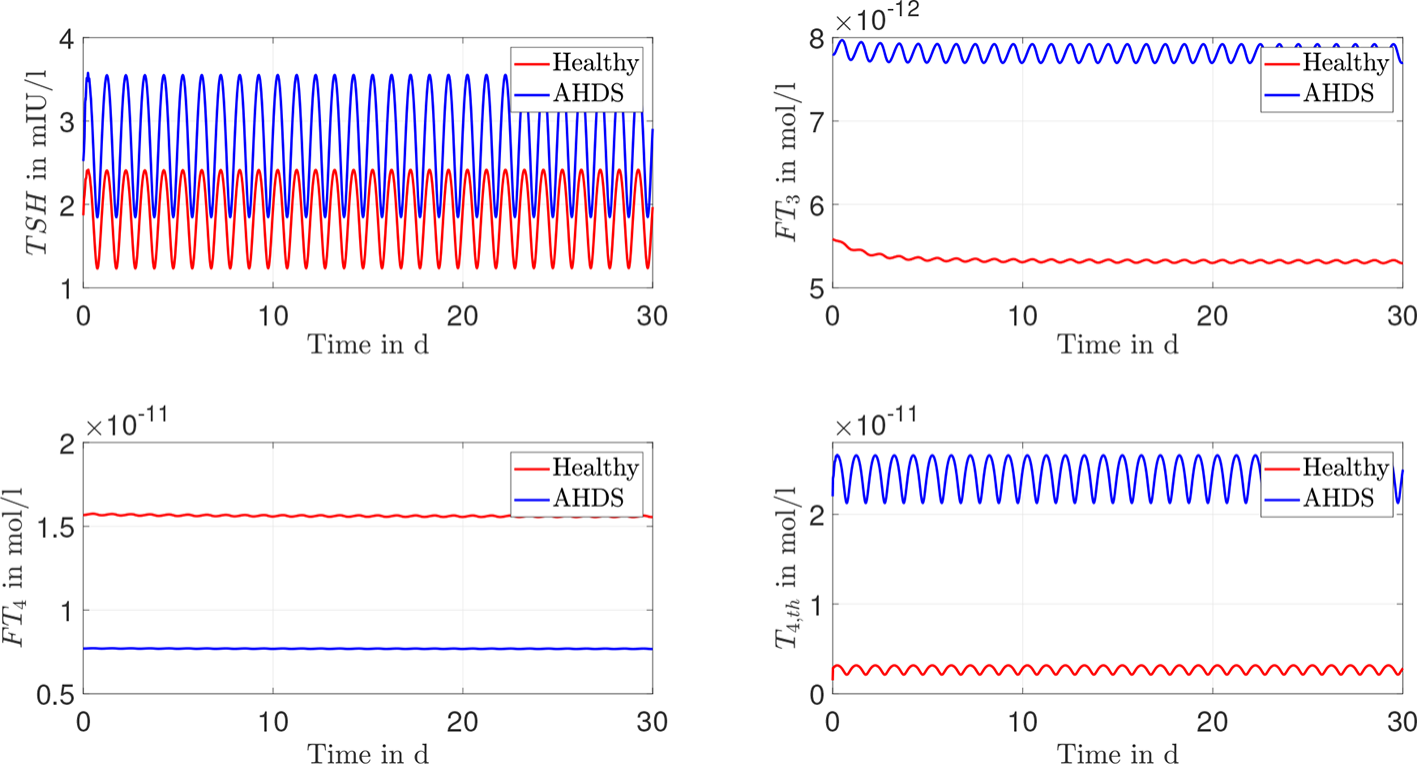
Results of the dynamic simulations of the linear modeling of the membrane transporter. Again, most of the numerical parameter values are based on (13, 15). The remaining parameters are estimated through a constrained parameter optimization approach and shown in **Table 2**.

Again, one can perform dynamic simulations with the model considering the linearly approximated membrane transporters and the mean value of the estimated parameters as indicated in **Table 2**. The courses of the hormone concentrations are given in **Figure 4**. One can see that there is virtually no difference in the dynamic course of the hormone concentrations between the Michaelis-Menten modeling of the membrane transporters and its linear approximation.

## Discussion

In this section, we discuss the obtained results and place them in a larger context. First, we focus on our main contribution, the investigations around the mechanisms of the AHDS. Second, we compare the Michaelis-Menten modeling approach to its linear approximation. Finally, we discuss the parameter estimation.

### Mechanisms of the AHDS

The documented results of the dynamic simulations (compare **Figures 3**, **4**) clearly demonstrate that the unusual hormone concentrations of AHDS patients are observed in the simulations of the mathematical model when damaged membrane transporters are considered. This result holds true for the Michaelis-Menten and the linear modeling of the membrane transporters. The simulated hormone concentrations of AHDS patients are additionally in line with the mean measured hormone concentrations presented by (2), which is a large study dealing with MCT8 deficiency (*TSH* = 2.97 mIU/l, *FT*_4_ = 9.48·10^-12^ mol/l).

One must also keep in mind that the hormone concentrations of healthy individuals should not change substantially, when incorporating membrane transporters. They should remain in the reference range of healthy individuals (*TSH*: 0.5 - 4.5 mIU/l, *FT*_4_: 1.2 – 2.7·10^-11^ mol/l and *FT*_3_: 3.5 – 6.3·10^-12^ mol/l (27)). The dynamic simulations of the mathematical model reveal (see **Figures 3**, **4**) that the hormone concentrations of healthy individuals remain within the reference range of healthy individuals. Thus, the introduction of the membrane transporters does not impact the usability of the mathematical model for healthy individuals. The incorporation of the membrane transporters extends the possible applications of the mathematical model.

Since the concentration of *T*_4,_ *_th_* does not correspond to a hormone concentration which can be measured with today’s assay technology, it is difficult to evaluate its accordance with human data. Alternatively, the concentration can be compared to studies that are made with mice, e.g., the study (8). In this study, an investigation is done regarding the thyroidal *T*_4_ content of Mct8 KO mice. It is reported that the thyroids of these mice contain approximately a 3-fold elevation of *T*_4_ compared to wild-type littermates (8). In the presented mathematical model one can interpret the state *T*_4,_ *_th_* on a high level as *T*_4_ content in thyroid cells. Regarding the Michaelis-Menten modeling approach of the membrane transporters and its linear approximation, the *T*_4_ content in thyroid cells (described by the state *T*_4,_ *_th_*) is approximately 18 times higher for AHDS patients compared to healthy individuals.

Therefore, the mathematical model is in line with the documented observation that the *T*_4_ content in thyroid cells is increased in MCT8 deficiency (8). This is an indication that the *T*_4_ content in thyroid cells is not only increased for Mct8 KO mice, but also for AHDS patients. Compared to the results of (8), one must keep in mind that the difference between the *T*_4_ content in thyroid cells of AHDS patients to healthy individuals is higher in the mathematical model (18-fold increase), than the documented difference of Mct8 KO mice to wild-type littermates (3-fold increase).

At that point we can additionally evaluate the hypothesis that the *T*_4_ retention in thyroid cells represents one important mechanism to the unusual hormone concentrations of AHDS patients (12). In the simulations of this work, damaged membrane transporters go along with an increased *T*_4_ content in thyroid cells and ultimately the unusual hormone concentrations of AHDS patients. Thus, additional evidence is given to the hypothesis stated in (12) by means of the mathematical model.

If we take a more precise look on the considerations mentioned in (12), one remarks a small but important difference between their considerations and our results. The authors in (12) assign the high concentration of *FT*_3_ to the retention of *T*_4_ inside the thyroid. In turn, the assumption is made that the low concentrations of *FT*_4_ are due to a renal contribution, as the renal *T*_4_ content is increased in MCT8 deficiency (9, 12).

The difference to our results is that the simulations lead to the entirety of the unusual hormone concentrations of AHDS patients, including a lower concentration of *FT*_4_. This is an indication that the *T*_4_ retention in thyroid cells does not only represent one mechanism leading to the unusual hormone concentrations of AHDS patients, but could even be fully responsible for these unusual concentrations. In other words, the simulations of the mathematical model suggest that an additional renal contribution might not be necessary to replicate the entirety of the unusual hormone concentrations of AHDS patients.

To evaluate the role of the kidney in the genesis of the unusual hormone concentrations of AHDS patients by means of the mathematical model, an explicit representation of it is necessary. This is currently not the case, because the model merges the effects of the different peripheral organs like the kidney and the liver as well as the effects of muscle tissue under one general component, the periphery. Once an explicit representation of the kidney is incorporated into the model, membrane transporters could be considered at the edge of the kidney to the bloodstream, which would allow a more precise investigation of the assumptions from (12). However, the further refinement of the model usually goes along with more parameters that must be estimated, which becomes more difficult. Nevertheless, the explicit consideration of the kidney in the mathematical model is an interesting issue for further research.

Finally, we discuss our results with respect to the single case report documented in (11) in which one AHDS patient was examined before and after thyroidectomy. Before thyroidectomy the patient received 75 (or 10 *µ*/g per day) of levothyroxine (*L*-*T*_4_) to normalize the *TSH* concentration. The exact hormone concentrations were *FT*_4_ ≈ 1.06·10^-11^ mol/l, *T*_3_ ≈ 7.92·10^-9^ mol/l and *TSH* =0.1 mIU/l [compare Figure 2 in (11)]. After thyroidectomy and 125 µg (or 6 µg/kg per day) of *L*-*T*_4_, the patient’s hormone concentrations were *FT*_4_ ≈ 1.05·10^-11^ mol/*l*, *T*_3_ ≈ 4.21·10^-9^ mol/*l* and *TSH* =0.48 mIU/l [again, compare Figure 2 in (11)].

Note that the normalization of the *TSH* concentration goes along with a substantially higher concentration of *T*_3_ before thyroidectomy compared to the concentration of *T*_3_ after thyroidectomy. This indicates that a retention of *T*_4_ in thyroid cells could be responsible for the high *T*_3_ concentrations, which is a conclusion in line with the simulation results. The *FT*_4_ concentrations remain approximately constant in both cases. As already mentioned, this points to extrathyroidal mechanisms explaining the low *FT*_4_ concentrations of AHDS patients. In contrast, the simulation results suggest that such extrathyroidal events do not have to be present in order to replicate the hormone concentrations, which seems to be a contradiction at first sight. However, since we do not consider the kidney explicitly in the mathematical model, we also do not exclude extrathyroidal mechanisms from possibly contributing to the unusual hormone concentrations. This aspect can currently not be answered by means of the model, since the kidney is not considered explicitly.

Furthermore, future work could focus on other phenomena related to MCT8 deficiency that were not treated in the context of this work. For example, in Mct8/D1 double KO mice, a partial normalization of thyroid hormone concentrations takes place [compare (28)]. An analysis whether the same observation can be made exploiting the mathematical model of the pituitary-thyroid feedback loop would potentially further deepen the knowledge regarding the AHDS.

### Comparison of the Michaelis-Menten modeling approach to its linear approximation

Two possibilities to model the membrane transporters are presented in this paper. The Michaelis-Menten modeling of the membrane transporters follows a common approach to model transporter/substrate reactions (29). A saturation of the transported *T*_4_ can take place, when no membrane transporters are available, i.e., *T*_4,_ *_th_* >>*K_MT_* in equation (2). The linear modeling of the membrane transporters has an appealing simplicity. The intuitive idea that a certain percentage (*k_l_*) of *T*_4_ is transported out of thyroid cells is easy to understand. However, this approximation is only applicable for a specific range of *T*_4,_ *_th_*, namely as long as *T*_4,_ *_th_* <<*K_MT_*. If this is not the case, the linear approximation is not valid anymore.

In our case, the necessary requirement in order to model the functionality of the membrane transporters linearly is fulfilled (*K_MT_* = 4.7·10^-6^ >> 2.50·10^-12^ = T_4,_ *_th_*). The results of the parameter estimation demonstrate that this approximation is meaningful. This can be seen in the following way. For healthy individuals the term *G_MT_*/*K_MT_* ≈ 0.43 of the Michaelis-Menten modeling is approximately equal to *k_l_* ≈ 0.43 of the linear modeling of the membrane transporters (compare **Tables 1**, **2**). The same holds true for the estimated parameters of AHDS patients: *G_MT_*/*K_MT_* ≈ 0.02, *k_l_* ≈ 0.02. Given these results, there might be no substantial advantage applying the Michaelis-Menten modeling of the membrane transporters over the linear modeling of the membrane transporters. In conclusion, the application of the linear modeling of the membrane transporters reduces the complexity of the model and leads to similar results.

The applied mathematical model exploits parameters that were determined for humans as well as parameters that were determined for rodents, even though some aspects of the thyroid homeostasis are different. The model does not aim for an exact representation of humans which would be impossible due to an inter-variability of the parameters even for humans only. However, note that it is possible to obtain an understanding of the cause-effect relationships even for these “generic” parameter values (that do not correspond to one individual human subject). Namely, the qualitative behavior that is obtained from simulating the model is the same for different parameter values. Mathematically, this can be shown by a sensitivity analysis for the parameters, compare (15). So even if the parameters do not all correspond to the true parameters of a (one individual) human subject, the observed phenomena are still representative for the cause-effect relationships in the human HPT axis.

So far, the loss of thyroid hormone transport activity is only considered at the thyroid gland for *T*_4_. Investigating how MCT8 deficiency affects the complete pituitary-thyroid feedback loop (i.e., considering a loss of thyroid hormone transport activity at further locations) is an interesting topic for future research.

### Parameter estimation

In the context of the parameter estimation, we considered the estimated values of *G_D_*_1_, *G_T_*, and *G_T3_* for healthy individuals as fixed for AHDS patients. This approach can be interpreted in a physiological sense that the biological maximal activity of D1 (corresponding to *G_D_*_1_ in the model) is the same for healthy individuals and AHDS patients. It is difficult to evaluate whether this approach is reasonable, since most studies only evaluate the *activity* of D1 and not the *maximal activity*. Additionally, the total D1 activity is an extensive parameter, i.e., it depends on the number of expressing cells, approximately on body mass. Therefore, results from cell culture experiments and expression data from biopsies cannot be readily translated to the organismal level.

For example, the activity of D1 inside the thyroid does not change for Mct8 KO mice compared to wild-type littermates (8). In turn, no results regarding the maximal activity exist. These observations motivated us to assume a constant maximal activity of D1 and constant values of *G_T3_* and *G_T_* in our parameter estimation, although there are no studies in the literature available examining this fact.

Furthermore, we consider dynamic hormone measurements of healthy individuals in order to calibrate our mathematical model. This improves the model in the sense that it approximates the dynamics of the real pituitary-thyroid feedback loop much better. Notice also that dynamic hormone measurements have so far not been used to estimate model parameters in any of the previous works related to the here applied model (13–15, 18).

## Conclusion

In this paper, we included membrane transporters in the mathematical model of the pituitary-thyroid feedback loop, originally developed by (13–15). The extended model fully replicates the unusual hormone concentrations of AHDS patients and suggests that the retention of *T*_4_ in thyroid cells could fully explain the unusual hormone concentrations of AHDS patients. Future work could focus on an explicit consideration of the kidney in the model in order to evaluate whether/how an accumulation of *T*_4_ in the kidney (in MCT8 deficiency) leads to the unusual hormone concentrations of AHDS patients.

## Data availability statement

The original contributions presented in the study are included in the article/**Supplementary Material**. Further inquiries can be directed to the corresponding author. The Matlab/Simulink files that were used to perform the simulations are available online https://doi.org/10.25835/0049176.

## Author contributions

TW drafted the manuscript and performed the calculations/simulations with Matlab/Simulink, under the supervision of MM and with input from JD. CV derived the Michaelis-Menten modeling approach of the membrane transporters and its linear approximation and worked on the incorporation of the membrane transporters into the mathematical model, also under the supervision of MM and with input of JD. All authors contributed to the article and approved the submitted version.

## Footnotes

1 Note that one could also sum up the net effects of D1 and D2 in a single term. Here, we do not follow this approach since it is less suitable to analyze, e.g., the effects of genetic variants [compare (18)] affecting the activity of D1 *or* D2 in the context of AHDS, which is an interesting subject for future research.

2 The maximal activity regarding the direct *T*_3_ synthesis path is denoted by G_T3_, compare **Figure 2**.

3 Note that by using this approach, we assume that the complete physiology (except for the introduced membrane transporters) is equal for AHDS patients and healthy individuals.
