# Supplementary material for: Mathematical modeling and simulation of thyroid homeostasis: Implications for the Allan-Herndon-Dudley syndrome
Source: Front Endocrinol (Lausanne). 2022 Dec 8;13:882788. doi: 10.3389/fendo.2022.882788 (PMC9772020; doi:10.3389/fendo.2022.882788)
Supplement: Supplementary file 1 [file DataSheet_1.pdf]

Supplementary Material: Mathematical Modeling and Simulation of Thyroid Homeostasis: Implications for the Allan-Herndon-Dudley Syndrome

Tobias M. Wolff *, **Carina Veil, Johannes W. Dietrich and Matthias A. Müller**

Correspondence*:
Tobias M. Wolff
wolff@irt.uni-hannover.de

S1 STATE OF THE ART: MATHEMATICAL MODEL OF THE PITUITARY-THYROID FEEDBACK LOOP

As mentioned in the main part of the paper, we want to present in detail the state of the art of the applied mathematical model of the pituitary-thyroid feedback loop. The block diagram of the mathematical model is illustrated in Figure 1.

First, a closer look is taken on the thyroid, which is represented by the grey block in the bottom left corner. In this block, one sees that the T_4 synthesis (bottom left corner of the “Thyroid” block) depends on TSH, the damping constant of TSH at the thyroid gland (D_T), the maximal secretory capacity of the thyroid gland (G_T) and the substrate concentrations of thyroglobulin (K_T) and iodide (K_I). The T_4 synthesis is modeled by means of the well known Michaelis-Menten kinetics. The underlying physiological process of the T_4 synthesis is of course much more complicated, since it additionally depends on, e.g., the activity of the thyroid peroxidase and sodium/iodide symporter. Nevertheless, a simplification of the underlying physiologic process is necessary in order to keep the model compact.

In the right-hand side of the T_4 synthesis block, dilution (denoted by α_{th}) and clearance (denoted by β_{th}) of T_4 in thyroid cells take place. Next, the concentration of $T_{4,th}$ is depicted, denoting the T_4 concentration in thyroid cells. One part of $T_{4,th}$ is directly transported out of thyroid cells. Here, we denote the maximal activity and the Michaelis-Menten constant of this transport process by G_{MT} and K_{MT}, respectively. The production of T_3 in thyroid cells functions through two pathways (3). The first pathway is a conversion of $T_{4,th}$ into T_3 by means of 5’-deiodinase type I (D1) and 5’-deiodinase type II (D2). This process is modeled via the maximal activities of D1 (G_{D1}), of D2 (G_{D2}) and the dissociation constants K_{M1}, K_{M2}, respectively. The second path is a direct synthesis of T_3 in thyroid cells, a process, again depending on the concentration of TSH, D_T and, additionally, the maximal activity of the T_3 synthesis path (G_{T3}). In order to account for diffusion related delays, we incorporate a dead time (τ_{03P}) concerning the T_3 that is produced in thyroid cells.

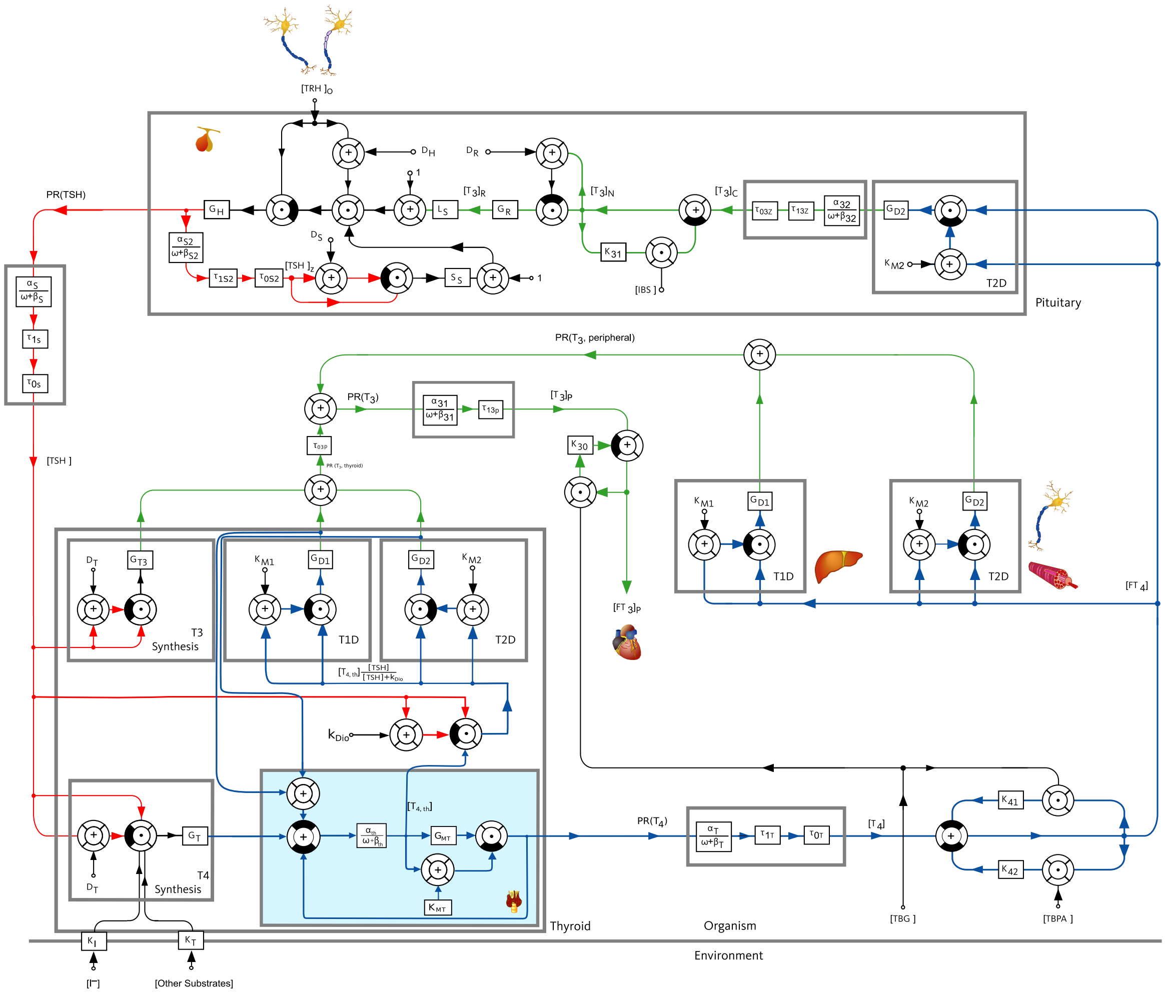

Figure 1. Block diagram of the pituitary-thyroid feedback loop including membrane transporters, extended from (1, 2, 3).

The entrance of T_4 in the blood stream is modeled as first order lag element, taking into account dilution (α_T) and clearance (β_T) of T_4. Furthermore, a dead time (τ_{0T}) is consider to account for diffusion processes. Additionally, the time constant of the first order lag element (τ_{1T}) is explicitly shown.

Only a small fraction of T_4 is available as FT_4, since most of it is bound to plasma binding proteins. This phenomenon is taken into account by considering the concentrations of thyroxine-binding globulin (TBG), transthyretin ($TBPA$), and their dissociation constants (K_{41}/K_{42}) as displayed in the bottom right corner.

The peripheral production of T_3 can be seen in the middle of Figure 1. The periphery summarizes the net effects of peripheral organs like the liver or the kidney. This means that we do not explicitly consider the liver and the kidney in the model. We rather summarize their net effects in the periphery. An explicit representation would certainly be helpful in several ways, e.g., to investigate whether the low T_4 concentrations of AHDS patients can be explained by an accumulation of T_4 in the kidney. However, an explicit representation would go along with new parameters for which numerical values are needed and new states that must be considered. This renders the model even more complex and induces more uncertainty, because the parameters would not necessarily be uniquely identifiable.

In these peripheral organs, D1 and D2 convert FT_4 into FT_3, a process which is again modeled by means of a Michaelis-Menten kinetics with G_{D1}, G_{D2}, K_{M1} and K_{M2}. This peripheral production is finalized by considering a first-order lag element, taking into account dilution (α_{31}) and clearance (β_{31}). The binding of T_3 to TBG and the respective dissociation constant (K_{41}) are also visible.

The production of T_3 in the pituitary is considered explicitly in the top right-hand corner of Figure 1. It depends on the maximal activity of D2 (G_{D2}), the respective dissociation constant (K_{M2}), the dilution (α_{32}), the clearance factor (β_{32}) and the dead time (τ_{3Z}). The central T_3 (T_{3z}) mainly serves as a feedback signal to the pituitary.

Inside the pituitary, which is illustrated in the top of the scheme, many different processes take place. One part of the T_{3z} binds to the intracellular-binding-substrate (IBS), which does not serve as a feedback signal. The damping constant D_R models the fact that T_3 must bind to specific thyroid hormone receptors in order to influence the concentration of TSH. The remaining constants (G_R and L_S) stand for the maximum gain of the pituitary receptors in relation to thyroid hormones and a breaking constant, respectively.

Furthermore, the influence of TRH is illustrated in the top of Figure 1. The receptors for TRH at the pituitary are taken into account with a damping constant (D_H). The ultra-short feedback loop of TSH on its own secretion (compare (2)) is considered with the respective dilution (α_{S2}) and clearance (β_{S2}) factors. The maximum secretory capacity of the pituitary (G_H) is shown. The entrance of TSH into the bloodstream is modeled with a first order lag element, with dilution (α_S) and clearance (β_S).

After having derived the relationships between the different hormone concentrations, one must find the numerical values of all introduced parameters. The parameter values used within this work are listed in Section S9 below. Ideally, we would like to use only human parameters. However, this is not possible for all parameters, consider, e.g., the damping constant of TRH at the pituitary D_H, which is not measurable in humans. Therefore, we partially exploit experimentally determined murine numerical parameter values. This results in a model which contains human and murine numerical parameter values. Nevertheless, this approach is meaningful for two reasons: first, there will always be a variation in the exact numerical parameters even for humans only. Second, when applying the mathematical model, we pursue the objective to get insight about the mechanisms of the AHDS, which is also possible when using murine and human numerical parameter values jointly. This is the case since (slightly) different parameter values lead to the same qualitative behavior of hormone concentrations in our model (compare also the sensitivity analysis in (3)).

S2 STATE TRANSFORMATION

Before stating the formal problem of the constrained parameter optimization for healthy individuals and AHDS patients in Sections S3 and S4, respectively, it is useful to introduce the system's differential

equations for the Michaelis-Menten modeling of the membrane transporters

$$\begin{aligned}\frac{dT_{4,th}}{dt}(t) =&\alpha_{th}\Bigg(G_T\frac{TSH(t)}{TSH(t)+D_T} - G_{MT}\frac{T_{4,th}(t)}{K_{MT}+T_{4,th}(t)} - G_{D1}\frac{T_{4,th}(t)\frac{TSH(t)}{TSH(t)+k_{Dio}}}{T_{4,th(t)}\frac{TSH(t)}{TSH(t)+k_{Dio}}+K_{M1}}\\ &- G_{D2}\frac{T_{4,th}(t)\frac{TSH(t)}{TSH(t)+k_{Dio}}}{T_{4,th}(t)\frac{TSH(t)}{TSH(t)+k_{Dio}}+K_{M2}}\Bigg) - \beta_{th}T_{4,th}(t)\end{aligned} \tag{S1}$$

$$\frac{dT_4}{dt}(t) =\alpha_T G_{MT}\frac{T_{4,th}(t-\tau_{0T})}{K_{MT}+T_{4,th}(t-\tau_{0T})} - \beta_T T_4(t) \tag{S2}$$

$$\begin{aligned}\frac{dT_{3p}}{dt}(t) =&\alpha_{31}\Bigg(G_{D1}\frac{FT_4(t)}{FT_4(t)+K_{M1}} + G_{D2}\frac{FT_4(t)}{FT_4(t)+K_{M2}} + G_{T3}\frac{TSH(t-\tau_{03P})}{D_T+TSH(t-\tau_{03P})}\\ &+ G_{D1}\frac{T_{4,th}(t-\tau_{03P})\frac{TSH(t-\tau_{03P})}{TSH(t-\tau_{03P})+k_{Dio}}}{T_{4,th}(t-\tau_{03P})\frac{TSH(t-\tau_{03P})}{TSH(t-\tau_{03P})+k_{Dio}}+K_{M1}}\\ &+ G_{D2}\frac{T_{4,th}(t-\tau_{03P})\frac{TSH(t-\tau_{03P})}{TSH(t-\tau_{03P})+k_{Dio}}}{T_{4,th}(t-\tau_{03P})\frac{TSH(t)}{TSH(t-\tau_{03P})+k_{Dio}}+K_{M2}}\Bigg) - \beta_{31}T_{3p}(t)\end{aligned} \tag{S3}$$

$$\frac{dT_{3c}}{dt}(t) =\alpha_{32}G_{D2}\frac{FT_4(t-\tau_{03Z})}{FT_4(t-\tau_{03Z})+K_{M2}} - \beta_{32}T_{3c}(t) \tag{S4}$$

$$\begin{aligned}\frac{dTSH}{dt}(t) =&\frac{\alpha_S G_H TRH(t-\tau_{0S})}{(TRH(t-\tau_{0S})+D_H)(1+S_S\frac{TSH_z(t-\tau_{0S})}{TSH_z(t-\tau_{0S})+D_S})(1+L_S G_R\frac{T_{3N}(t-\tau_{0S})}{T_{3N}(t-\tau_{0S})+D_R})}\\ &- \beta_S TSH(t)\end{aligned} \tag{S5}$$

$$\begin{aligned}\frac{dTSH_z}{dt}(t) =&\frac{\alpha_{S2} G_H TRH(t-\tau_{0S2})}{(TRH(t-\tau_{0S2})+D_H)(1+S_S\frac{TSH_z(t-\tau_{0S2})}{TSH_z(t-\tau_{0S2})+D_S})(1+L_S G_R\frac{T_{3N}(t-\tau_{0S2})}{T_{3N}(t-\tau_{0S2})+D_R})}\\ &- \beta_{S2} TSH_z(t)\end{aligned} \tag{S6}$$

with the relationships

$$FT_3 = T_{3p}\frac{1}{1+K_{30}TBG} \tag{S7}$$

$$FT_4 = T_4\frac{1}{1+K_{41}TBG+K_{42}TBPA} \tag{S8}$$

$$T_{3N} = T_{3c}\frac{1}{1+K_{31}IBS} \tag{S9}$$

$$TRH(t) = TRH_0(1+0.6cos(2\pi(t/86400))) \tag{S10}$$

For the linear modeling of the membrane transporters, one must replace the term

$$G_{MT}\frac{T_{4,th}}{K_{MT}+T_{4,th}} \tag{S11}$$

in equations (S1) and (S2) by

$$k_l T_{4,th}. \tag{S12}$$

As mentioned in the main part of this paper, we perform a constrained parameter optimization in order to identify the parameters G_T, G_{D1}, G_{T3} and G_{MT} (or k_l). In other words, we are looking for the configuration of parameters which fits best the given real measured hormone data under the condition that the (steady-state or dynamic) differential equations (S1) - (S6) are satisfied.

One challenge of this approach is that the order of magnitude of the differential equations is very different. The value of FT_3 is of order 10^{-12}, whereas the order of TSH is 1. In order to obtain a numerically well conditioned problem, we hence perform the following state transformation

$$\begin{pmatrix} \widetilde{T_{4,th}} \\ \widetilde{T_4} \\ \widetilde{T_{3p}} \\ \widetilde{T_{3c}} \\ \widetilde{TSH} \\ \widetilde{TSH_z} \end{pmatrix} = \mathbf{z} = \mathbf{Tx} = \mathbf{T}\begin{pmatrix} T_{4,th} \\ T_4 \\ T_{3p} \\ T_{3c} \\ TSH \\ TSH_z \end{pmatrix}, \tag{S13}$$

with

$$\mathbf{T} = \begin{pmatrix} 10^{12} & 0 & 0 & 0 & 0 & 0 \\ 0 & 10^{11} & 0 & 0 & 0 & 0 \\ 0 & 0 & 10^{12} & 0 & 0 & 0 \\ 0 & 0 & 0 & 10^{8} & 0 & 0 \\ 0 & 0 & 0 & 0 & 1 & 0 \\ 0 & 0 & 0 & 0 & 0 & 1 \end{pmatrix}. \tag{S14}$$

This transformation matrix leads to (transformed) hormone concentrations in the identification (FT_3, FT_4, $T_{4,th}$ and TSH) that are in the order of magnitude of 10^0. The dynamics of the transformed system are

$$\dot{\mathbf{z}} = \mathbf{T}\dot{\mathbf{x}} = \mathbf{T}\mathbf{f}(\mathbf{x}) = \mathbf{T}\mathbf{f}(\mathbf{T}^{-1}\mathbf{z}), \tag{S15}$$

where $\mathbf{f}(\mathbf{x})$ denotes the right hand side of the differential equations (S1) - (S6) and x, z as defined in (S13). When the mentioned values are plugged in, the expression

$$\dot{\mathbf{z}} = \begin{pmatrix} 10^{12} & 0 & 0 & 0 & 0 & 0 \\ 0 & 10^{11} & 0 & 0 & 0 & 0 \\ 0 & 0 & 10^{12} & 0 & 0 & 0 \\ 0 & 0 & 0 & 10^{8} & 0 & 0 \\ 0 & 0 & 0 & 0 & 1 & 0 \\ 0 & 0 & 0 & 0 & 0 & 1 \end{pmatrix} \mathbf{f} \begin{pmatrix} 10^{-12}\widetilde{T_{4,th}} \\ 10^{-11}\widetilde{T_4} \\ 10^{-12}\widetilde{T_{3p}} \\ 10^{-8}\widetilde{T_{3c}} \\ \widetilde{TSH} \\ \widetilde{TSH_z} \end{pmatrix} \tag{S16}$$

is obtained. The transformed differential equations are

$$\frac{d\widetilde{T_{4,th}}}{dt}(t) = 10^{12}\Bigg(\alpha_{th}\Bigg(G_T\frac{\widetilde{TSH}(t)}{\widetilde{TSH}(t)+D_T} - 10^{-6}\widetilde{G_{MT}}\frac{10^{-12}\widetilde{T_{4,th}}(t)}{K_{MT}+10^{-12}\widetilde{T_{4,th}}(t)}$$
$$- 10^{-8}\widetilde{G_{D1}}\frac{10^{-12}\widetilde{T_{4,th}}(t)\frac{\widetilde{TSH}(t)}{\widetilde{TSH}(t)+k_{Dio}}}{10^{-12}\widetilde{T_{4,th}}(t)\frac{\widetilde{TSH}(t)}{\widetilde{TSH}(t)+k_{Dio}}+K_{M1}} - G_{D2}\frac{10^{-12}\widetilde{T_{4,th}}(t)\frac{\widetilde{TSH}(t)}{\widetilde{TSH}(t)+k_{Dio}}}{10^{-12}\widetilde{T_{4,th}}(t)\frac{\widetilde{TSH}(t)}{\widetilde{TSH}(t)+k_{Dio}}+K_{M2}}\Bigg)$$
$$- \beta_{th}T_{4,th}(t)\Bigg) \tag{S17}$$

$$\frac{d\widetilde{T_4}}{dt}(t) = 10^{11}\Bigg(\alpha_T\, 10^{-6}\widetilde{G_{MT}}\frac{10^{-12}\widetilde{T_{4,th}}(t-\tau_{0T})}{K_{MT}+10^{-12}\widetilde{T_{4,th}}(t-\tau_{0T})} - \beta_T 10^{-11}\widetilde{T_4}(t)\Bigg) \tag{S18}$$

$$\frac{d\widetilde{T_{3p}}}{dt}(t) = 10^{12}\Bigg(\alpha_{31}\Bigg(10^{-8}\widetilde{G_{D1}}\frac{10^{-11}\widetilde{FT_4}(t)}{10^{-11}\widetilde{FT_4}(t)+K_{M1}} + G_{D2}\frac{10^{-11}\widetilde{FT_4}(t)}{10^{-11}\widetilde{FT_4}(t)+K_{M2}}$$
$$+ 10^{-8}\widetilde{G_{D1}}\frac{10^{-12}\widetilde{T_{4,th}}(t-\tau_{03P})\frac{\widetilde{TSH}(t-\tau_{03P})}{\widetilde{TSH}(t-\tau_{03P})+k_{Dio}}}{10^{-12}\widetilde{T_{4,th}}(t-\tau_{03P})\frac{\widetilde{TSH}(t-\tau_{03P})}{\widetilde{TSH}(t-\tau_{03P})+k_{Dio}}+K_{M1}}$$
$$+ G_{D2}\frac{10^{-12}\widetilde{T_{4,th}}(t-\tau_{03P})\frac{\widetilde{TSH}(t-\tau_{03P})}{\widetilde{TSH}(t-\tau_{03P})+k_{Dio}}}{10^{-12}\widetilde{T_{4,th}}(t-\tau_{03P})\frac{\widetilde{TSH}(t-\tau_{03P})}{\widetilde{TSH}(t-\tau_{03P})+k_{Dio}}+K_{M2}}$$
$$+ 10^{-14}\widetilde{G_{T3}}\frac{\widetilde{TSH}(t-\tau_{03P})}{D_T+\widetilde{TSH}(t-\tau_{03P})}\Bigg) - \beta_{31}10^{-12}\widetilde{T_{3p}(t)}\Bigg) \tag{S19}$$

$$\frac{d\widetilde{T_{3c}}}{dt}(t) = 10^{8}\Bigg(\alpha_{32}G_{D2}\frac{10^{-11}\widetilde{FT_4}(t-\tau_{03Z})}{10^{-11}\widetilde{FT_4}(t-\tau_{03Z})+K_{M2}} - \beta_{32}10^{-8}\widetilde{T_{3c}}(t)\Bigg) \tag{S20}$$

$$\frac{d\widetilde{TSH}}{dt}(t) = \frac{\alpha_S G_H TRH(t-\tau_{0S})}{(TRH(t-\tau_{0S})+D_H)\left(1+S_S\frac{\widetilde{TSH_z}(t-\tau_{0S})}{\widetilde{TSH_z}(t-\tau_{0S})+D_S}\right)\left(1+L_S G_R\frac{10^{-8}\widetilde{T_{3N}}(t-\tau_{0S})}{10^{-8}\widetilde{T_{3N}}(t-\tau_{0S})+D_R}\right)}$$
$$- \beta_S\widetilde{TSH}(t) \tag{S21}$$

$$\frac{d\widetilde{TSH_z}}{dt}(t) = \frac{\alpha_{S2} G_H TRH(t-\tau_{0S2})}{(TRH(t-\tau_{0S2})+D_H)\left(1+S_S\frac{\widetilde{TSH_z}(t-\tau_{0S2})}{\widetilde{TSH_z}(t-\tau_{0S2})+D_S}\right)\left(1+L_S G_R\frac{10^{-8}\widetilde{T_{3N}}(t-\tau_{0S2})}{10^{-8}\widetilde{T_{3N}}(t-\tau_{0S2})+D_R}\right)}$$
$$- \beta_{S2}\widetilde{TSH_z}(t)\,. \tag{S22}$$

Furthermore, the relationships of the hormone concentrations

$$FT_3 = 10^{-12}\widetilde{T_{3p}}\frac{1}{1+K_{30}TBG} = 10^{-12}\widetilde{FT_3} \tag{S23}$$

$$\widetilde{FT_3} = \widetilde{T_{3p}}\frac{1}{1+K_{30}TBG} \tag{S24}$$

$$FT_4 = 10^{-11}\widetilde{T_4}\frac{1}{1+K_{41}\,TBG+K_{42}\,TBPA} = 10^{-11}\widetilde{FT_4} \tag{S25}$$

$$\widetilde{FT_4} = \widetilde{T_4}\frac{1}{1+K_{41}\,TBG+K_{42}\,TBPA} \tag{S26}$$

$$T_{3N} = 10^{-8}\widetilde{T_{3c}}\frac{1}{1+K_{31}IBS} = 10^{-8}\widetilde{T_{3N}} \tag{S27}$$

$$\widetilde{T_{3N}} = \widetilde{T_{3c}}\frac{1}{1+K_{31}IBS} \tag{S28}$$

need to be considered. Using this state transformation, all variables are in the same order of magnitude.

S3 PARAMETER ESTIMATION FOR HEALTHY INDIVIDUALS USING DYNAMIC HORMONE MEASUREMENTS

As mentioned in the main part, we here explain the parameter estimation for healthy individuals using measured mean dynamic hormone concentrations $FT_{3,\text{meas}}(t)$, $FT_{4,\text{meas}}(t)$, and $TSH_{\text{meas}}(t)$. To this end, we use the dynamic hormone concentrations of healthy individuals documented in (4). The concept is to minimize the normalized quadratic error between measured (dynamic) hormone concentrations and simulated hormone concentrations. In other words, we want to find the configuration of the G_T, G_{D1}, G_{T3}, and G_{MT} parameters that explains best the given measured (dynamic) hormone concentrations. In mathematical terms, we minimize the objective function

$$\begin{aligned} J(G_T, G_{D1}, G_{T3}, G_{MT}) = &\sum_{t=0}^{15\cdot 24\text{h}}\Big(\frac{FT_{3,\text{meas}}(t) - FT_{3,\text{model}}(t)}{\overline{FT}_{3,\text{meas}}}\Big)^2 + \sum_{t=0}^{15\cdot 24\text{h}}\Big(\frac{FT_{4,\text{meas}}(t) - FT_{4,\text{model}}(t)}{\overline{FT}_{4,\text{meas}}}\Big)^2 \\ &+ \sum_{t=0}^{15\cdot 24\text{h}}\Big(\frac{TSH_{\text{meas}}(t) - TSH_{\text{model}}(t)}{\overline{TSH}_{\text{meas}}}\Big)^2 \end{aligned} \tag{S29}$$

where $FT_{3,\text{model}}(t)$, $FT_{4,\text{model}}(t)$, and $TSH_{\text{model}}(t)$ are the (back-transformed) numerical solutions to the transformed system of differential equations (S17) - (S22)[1] with the constraints that $G_T \geq 0$, $G_{D1} \geq 0$, $G_{T3} \geq 0$, and $G_{MT} \geq 0$. The constants $\overline{FT}_{3,\text{meas}}$, $\overline{FT}_{4,\text{meas}}$ and $\overline{TSH}_{\text{meas}}$ are the mean hormone concentrations of all patients *and* all time points. The cost function sums up the normalized quadratic difference lasting 15 days. To generate dynamic hormone profiles lasting 15 days, we duplicate the 24 hours profile, see (4), 15 times. This longer hormone profile is necessary to guarantee that the transient and

[1] For the estimation, we needed to set the delays of the system of differential equations to zero since Matlab does not provide a solver for stiff *and* delayed differential equations. We aimed to solve the system of delayed differential equations with the built-in function $dde23$. However, the solver needed such a small step size that takes an extremely long time to solve the system of delayed differential equations. Furthermore, the dead times are rather low (≤ 1 hour) meaning that these dead times do not influence the dynamics considerably.

the steady-state behavior is considered in the estimation. This approach implies the assumption that the measured dynamic hormone concentrations remain unchanged throughout 15 days.

After solving this optimization problem, we observed the following: different initial guesses of the optimization problem lead to different minima with the same cost. Consequently, there are different parameter configurations of G_T, G_{D1}, G_{T3} and G_{MT} that all explain equally well the given dynamic hormone measurements. This problem was also observed, when only steady-state hormone measurements are used for the identification of parameters of the model, compare (3). To solve this problem, we incorporate the information into the cost function that 20 % of T_3 is produced inside the thyroid gland and the remaining 80 % in peripheral organs, according to (5, 6)[2]. This is realized by penalizing the difference to this relation in the cost function. On the right hand side of (S29), we add $200|0.8 - \mathrm{PR}_{\mathrm{peri}}(t)|^2$, where $\mathrm{PR}_{\mathrm{peri}}(t)$ is defined as the part of T_3 which is produced in the periphery, i.e.,

$$\mathrm{PR}_{\mathrm{peri}}(t) = \frac{P_{\mathrm{peri}}(t)}{P_{\mathrm{total}}(t)} \tag{S30}$$

with

$$P_{\mathrm{peri}}(t) = G_{D1}\frac{FT_4(t)}{FT_4(t) + K_{M1}} + G_{D2}\frac{FT_4(t)}{FT_4(t) + K_{M2}} \tag{S31}$$

and

$$\begin{aligned} P_{\mathrm{total}}(t) =& G_{D1}\frac{FT_4(t)}{FT_4(t) + K_{M1}} + G_{D2}\frac{FT_4(t)}{FT_4(t) + K_{M2}} + G_{D1}\frac{T_{4,th}(t-\tau_{03P})\frac{TSH(t-\tau_{03P})}{TSH(t-\tau_{03P})+k_{Dio}}}{T_{4,th}(t-\tau_{03P})\frac{TSH(t-\tau_{03P})}{TSH(t-\tau_{03P})+k_{Dio}} + K_{M1}} \\ &+ G_{D2}\frac{T_{4,th}(t-\tau_{03P})\frac{TSH(t-\tau_{03P})}{TSH(t-\tau_{03P})+k_{Dio}}}{T_{4,th}(t-\tau_{03P})\frac{TSH(t-\tau_{03P})}{TSH(t-\tau_{03P})+k_{Dio}} + K_{M2}} + G_{T3}\frac{TSH(t-\tau_{03P})}{D_T + TSH(t-\tau_{03P})}. \end{aligned} \tag{S32}$$

Hence, the updated cost function is

$$\begin{aligned} J(G_T, G_{D1}, G_{T3}, G_{MT}) =& \sum_{t=0}^{15\cdot 24\mathrm{h}} \Big(\frac{FT_{3,\mathrm{meas}}(t) - FT_{3,\mathrm{model}}(t)}{\overline{FT}_{3,\mathrm{meas}}}\Big)^2 + \sum_{t=0}^{15\cdot 24\mathrm{h}} \Big(\frac{FT_{4,\mathrm{meas}}(t) - FT_{4,\mathrm{model}}(t)}{\overline{FT}_{4,\mathrm{meas}}}\Big)^2 \\ &+ \sum_{t=0}^{15\cdot 24\mathrm{h}} \Big(\frac{TSH_{\mathrm{meas}}(t) - TSH_{\mathrm{model}}(t)}{\overline{TSH}_{\mathrm{meas}}}\Big)^2 + \sum_{t=0}^{15\cdot 24\mathrm{h}} 200|0.8 - \mathrm{PR}_{\mathrm{peri}}(t)|^2. \end{aligned} \tag{S33}$$

Since the remaining production of T_3 will necessarily take place in thyroid cells (compare eq. (S3)), we do not need to incorporate a term related to the production of T_3 in thyroid cells. Using this additional information, we obtain unique optimal parameter values.

After having determined the unique optimal parameter configuration, we simulate the hormone concentrations 100 times and artificially corrupt the simulated concentrations by some noise following a normal distribution with $\mu = 0$ and $\sigma = 0.1$. For each dataset, we estimate the parameters applying the aforementioned procedure. The results are shown in Table 1 of the main part.

[2] Note that this relation was also used to calibrate the applied mathematical model in (3).

Further, to quantify the uncertainty of the parameter estimation by means of individual hormone measurements, we determine the parameters G_T, G_{D1}, G_{T3} and G_{MT} individually for 27 different healthy individuals, documented in (4). This individual estimation leads to the results presented in Table S1 and Section S5.

The estimation related to the linear modeling of the membrane transporters works completely analogous. One must simply replace the $G_{MT}\frac{T_{4,th}}{K_{MT}+T_{4,th}}$ term by $k_l T_{4,th}$ and estimate k_l instead of G_{MT}. The results are presented in Table 2 of the main part and Table S3 (regarding the individual parameter estimation).

S4 PARAMETER ESTIMATION FOR AHDS PATIENTS USING STATIC HORMONE CONCENTRATIONS

As mentioned in the main part of this paper, we perform a constrained parameter optimization using mean steady-state hormone concentrations in order to estimate the parameter G_{MT} (or k_l) for AHDS patients. In other words, we are looking for the configuration of parameters which fits best the given real measured hormone data under the condition that the steady state equations are satisfied. In formal words, the task is to minimize the objective function

$$J(G_{MT}) = \left(\frac{\overline{FT}_{3,\text{meas}} - FT_{3,\text{model}}}{\overline{FT}_{3,\text{meas}}}\right)^2 + \left(\frac{\overline{TSH}_{\text{meas}} - TSH_{\text{model}}}{\overline{TSH}_{\text{meas}}}\right)^2 + \left(\frac{\overline{FT}_{4,\text{meas}} - FT_{4,\text{model}}}{\overline{FT}_{4,\text{meas}}}\right)^2 \tag{S34}$$

subject to the nonlinear equality constraints

$$\begin{aligned} 0 = \alpha_{th}\Bigg(& G_T\frac{TSH}{TSH+D_T} - G_{MT}\frac{T_{4,th}}{K_{MT}+T_{4,th}} - G_{D1}\frac{T_{4,th}\frac{TSH}{TSH+k_{Dio}}}{T_{4,th}\frac{TSH}{TSH+k_{Dio}}+K_{M1}} \\ & - G_{D2}\frac{T_{4,th}\frac{TSH}{TSH+k_{Dio}}}{T_{4,th}\frac{TSH}{TSH+k_{Dio}}+K_{M2}}\Bigg) - \beta_{th}T_{4,th} \end{aligned} \tag{S35}$$

$$0 = FT_4 - \frac{1}{1+K_{41}\,TBG+K_{42}\,TBPA}\frac{\alpha_T}{\beta_T}G_{MT}\frac{T_{4,th}}{K_{MT}+T_{4,th}} \tag{S36}$$

$$\begin{aligned} 0 = FT_3 - \frac{1}{1+K_{30}TBG}\frac{\alpha_{31}}{\beta_{31}}\Bigg(& G_{D1}\frac{FT_4}{FT_4+K_{M1}} + G_{D2}\frac{FT_4}{FT_4+K_{M2}} + G_{D1}\frac{T_{4,th}\frac{TSH}{TSH+k_{Dio}}}{T_{4,th}\frac{TSH}{TSH+k_{Dio}}+K_{M1}} \\ & + G_{D2}\frac{T_{4,th}\frac{TSH}{TSH+k_{Dio}}}{T_{4,th}\frac{TSH}{TSH+k_{Dio}}+K_{M2}} + G_{T3}\frac{TSH}{D_T+TSH}\Bigg) \end{aligned} \tag{S37}$$

$$\begin{aligned} 0 = TSH^2\left(G_{MT}\frac{T_{4,th}}{K_{MT}+T_{4,th}}q_1+q_2\right) + TSH\left(G_{MT}\frac{T_{4,th}}{K_{MT}+T_{4,th}}q_3+q_4-p_9\right) \\ - G_{MT}\frac{T_{4,th}}{K_{MT}+T_{4,th}}p_{10} - p_{11} \end{aligned} \tag{S38}$$

Table S1. Statistics of the Parameter estimation

Parameter	Healthy Individuals G_{D1} in $10^{-8}\frac{\mathrm{mol}}{\mathrm{s}}$	G_{T3} in $10^{-13}\frac{\mathrm{mol}}{\mathrm{s}}$	G_T in $10^{-12}\frac{\mathrm{mol}}{\mathrm{s}}$	G_{MT} in $10^{-6}\frac{\mathrm{mol}}{\mathrm{s}}$	AHDS Patients G_{MT} in $10^{-6}\frac{\mathrm{mol}}{\mathrm{s}}$
Mean	2.5113	1.7936	3.5698	2.0913	0.1843*
Median	2.5873	0.2437	3.4418	1.9614	0.2043
Standard deviation	0.5885	3.6397	0.7846	0.5079	0.1234
Coefficient of variation	0.2343	2.0293	0.2198	0.2429	0.6693

The * symbol designates statistical significance.

and the following bounds

$$G_{MT} \geq 0 \tag{S39}$$

$$FT_3 \geq 0 \tag{S40}$$

$$FT_4 \geq 0 \tag{S41}$$

$$TSH \geq 0 \tag{S42}$$

$$T_{4,th} \geq 0. \tag{S43}$$

The bounds are meaningful given that the parameter G_{MT} as well as the hormone concentrations physiologically only make sense when they are positive. We only deal with steady-state expressions, therefore, the time dependence of the variables is neglected. Note that we plugged in the steady state expressions of the differential equations of T_{3c} and of TSH_z into the steady state expression of TSH. This leads to condition (S38). The detailed steps how to reach (S38), which are cumbersome but straightforward, are shown in Section S8 for completeness. The results are presented in Table 1 of the main part.

We solve this optimization problem for the mean steady-state hormone concentrations of 13 different AHDS patients given in (7, 8, 9, 10, 11). This enables us to perform parametric bootstrapping. After having determined the optimal value of G_{MT}, we simulate the hormone concentrations and corrupt the FT_3, FT_4 and TSH concentrations by some noise following a normal distribution with $\mu = 0$ and $\sigma = 0.1$. In this way, we generate *dynamic* hormone concentrations of AHDS patients. Therefore, the optimal parameters of the generated hormone concentrations are determined by the procedure outlined in Section S3 with the only difference that we need to estimate only G_{MT} and not additionally G_{D1}, G_T, and G_{T3}.

S5 UNCERTAINTY QUANTIFICATION BASED ON INDIVIDUALLY ESTIMATED PARAMETERS

In this section, we show the results regarding the quantification of the uncertainty of the estimated parameters when these are estimated individually using the approach explained in Sections S3 and S4. First, we show the results for the Michaelis-Menten modeling of the membrane transporters and, second, we show the analogous results for the linear modeling of the membrane transporters.

Table S2. Associated Steady-State Hormone Concentrations

Hormone Concentration	FT_3 in $10^{-12}\frac{\mathrm{mol}}{\mathrm{L}}$	FT_4 in $10^{-11}\frac{\mathrm{mol}}{\mathrm{L}}$	TSH in $\frac{\mathrm{mIU}}{\mathrm{L}}$	$T_{4,th}$ in $10^{-12}\frac{\mathrm{mol}}{\mathrm{L}}$
	Healthy Individuals			
Mean	5.56	1.74	1.85	3.20
Median	5.55	1.68	1.87	3.21
Standard deviation	0.61	0.28	0.16	1.13
Coefficient of variation	0.11	0.16	0.09	0.35
	AHDS Patients			
Mean	8.27*	1.02*	2.56*	25.46*
Median	7.53	1.18	2.27	20.61
Standard deviation	1.34	0.31	0.48	9.64
Coefficient of variation	0.16	0.30	0.19	0.38

The * symbol designates statistical significance.

S5.1 Michaelis-Menten Modeling

The results of the uncertainty quantification of the G_{D1}, G_{T3}, G_T and G_{MT} are shown in Table S1. In general, the uncertainty quantification based on individually estimated parameters yields similar results as the uncertainty quantification based on bootstrapping in the main part. Except for the G_{T3} parameter, the numerical values of the parameters are not subject to large uncertainties. Here, we can additionally show that the difference in the mean of the G_{MT} parameter is significant (based on a two-sample t-test with a significance level of 5 %).

Next, the computed steady-state hormone concentrations are illustrated in Table S2. One can observe that the hormone concentrations of healthy individuals and of AHDS patients are not subject to large variations. All hormone concentrations have a coefficient of variation which is below 40 %. On one side, we observe the characteristic hormone concentrations of AHDS patients. The mean FT_3 concentration is 49 % times higher, the mean FT_4 concentration is 41 % lower, and the mean TSH concentration is 38 % higher for AHDS patients compared to healthy individuals. Interestingly, the mean $T_{4,th}$ concentration is approximately 800 % higher for AHDS patients compared to healthy individuals. Moreover, the mean hormone concentrations differ significantly from healthy individuals to AHDS patients (again based on a two-sample t-test with a significance level of 5 %).

Finally, as in the main part, we simulate the hormone concentrations of healthy individuals and AHDS patients, as shown in Figure 2, using the mean parameters of Table S1. Since the mean parameters are similar to the one determined using bootstrapping, the hormone concentrations in Figure 2 are similar to the ones shown in Figure 3 of the main part.

S5.2 Linear Modeling

In this section, we consider the linear modeling of the membrane transporters. As visible in Table S3, the uncertainty quantification of the parameters yields once again similar results compared to the application of bootstrapping, compare Table 2 of the main part. The mean values of the FT_3, FT_4, TSH and $T_{4,th}$

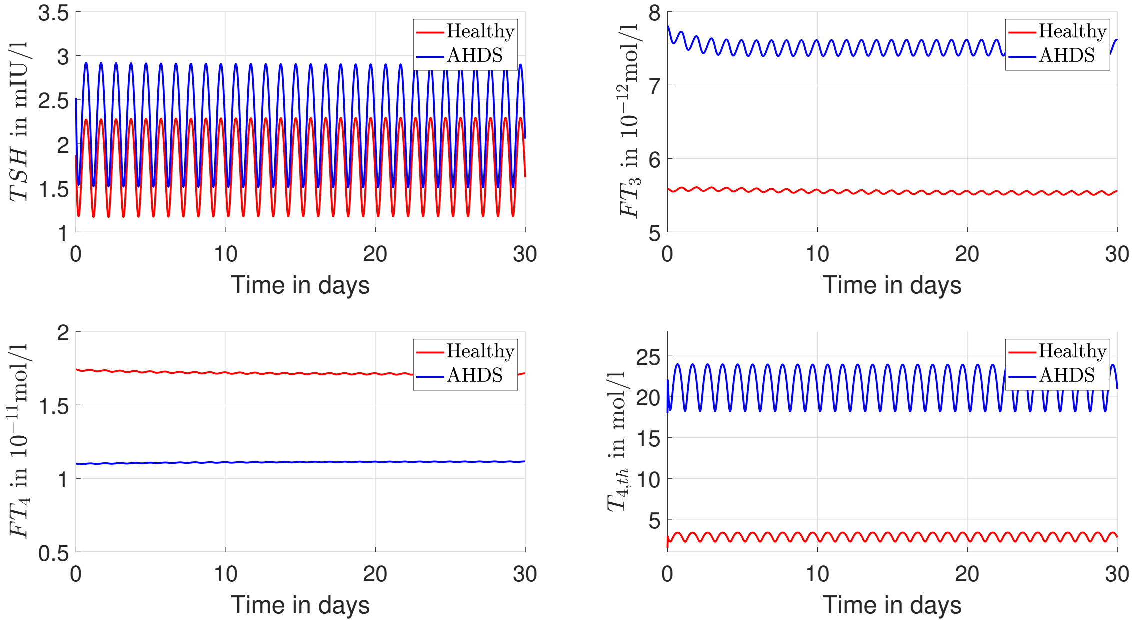

Figure 2. Results of the dynamic simulations for the Michaelis-Menten modeling of the membrane transporters. Most of the numerical parameter values are based on the suggestions of (1, 3), compare Section S9. The remaining unknown parameters of the model are estimated through a constrained parameter optimization and shown in Table S1.

Table S3. Statistics of the Parameter estimation

Parameter	Healthy Individuals				AHDS Patients
	G_{D1} in $10^{-8}\frac{\text{mol}}{\text{s}}$	G_{T3} in $10^{-13}\frac{\text{mol}}{\text{s}}$	G_T in $10^{-12}\frac{\text{mol}}{\text{s}}$	k_l in $\frac{1}{\text{s}}$	k_l in $\frac{1}{\text{s}}$
Mean	2.4909	1.9371	3.5671	0.4451	0.0392*
Median	2.5866	0.3865	3.4416	0.4173	0.0434
Standard deviation	0.6135	3.9763	0.7876	0.1083	0.0262
Coefficient of variation	0.2463	2.0527	0.2208	0.2433	0.6692

The * symbol designates statistical significance.

concentrations do not differ considerably from the Michaelis-Menten modeling approach. In Table S4, we observe once again the characteristic hormone concentrations of AHDS patients, a (46 %) higher FT_3 and a (36 %) higher TSH concentration together with a (40 %) lower FT_4 concentration compared to healthy individuals. The $T_{4,th}$ concentration is approximately 800 % for AHDS patients compared to healthy individuals. Finally, in Figure 3, the simulation results of the dynamic hormone concentrations are shown.

In conclusion, we want to point out that we applied two different approaches to quantify the uncertainty (bootstrapping and individual parameter estimation) of the parameters and that both yielded similar results in terms of how uncertain which parameters are.

Table S4. Associated Steady-State Hormone Concentrations

Hormone Concentration	Healthy Individuals*			
	FT_3 in $10^{-12}\frac{\text{mol}}{\text{L}}$	FT_4 in $10^{-11}\frac{\text{mol}}{\text{L}}$	TSH in $\frac{\text{mIU}}{\text{L}}$	$T_{4,th}$ in $10^{-12}\frac{\text{mol}}{\text{L}}$
Mean	5.56	1.74	1.85	3.20
Median	5.55	1.68	1.87	3.20
Standard deviation	0.61	0.28	0.16	1.13
Coefficient of variation	0.11	0.16	0.09	0.35
	AHDS Patients			
Mean	8.14*	1.05*	2.51*	24.73*
Median	7.52	1.18	2.27	20.66
Standard deviation	1.29	0.30	0.45	9.32
Coefficient of variation	0.16	0.28	0.18	0.38

The * symbol designates statistical significance.

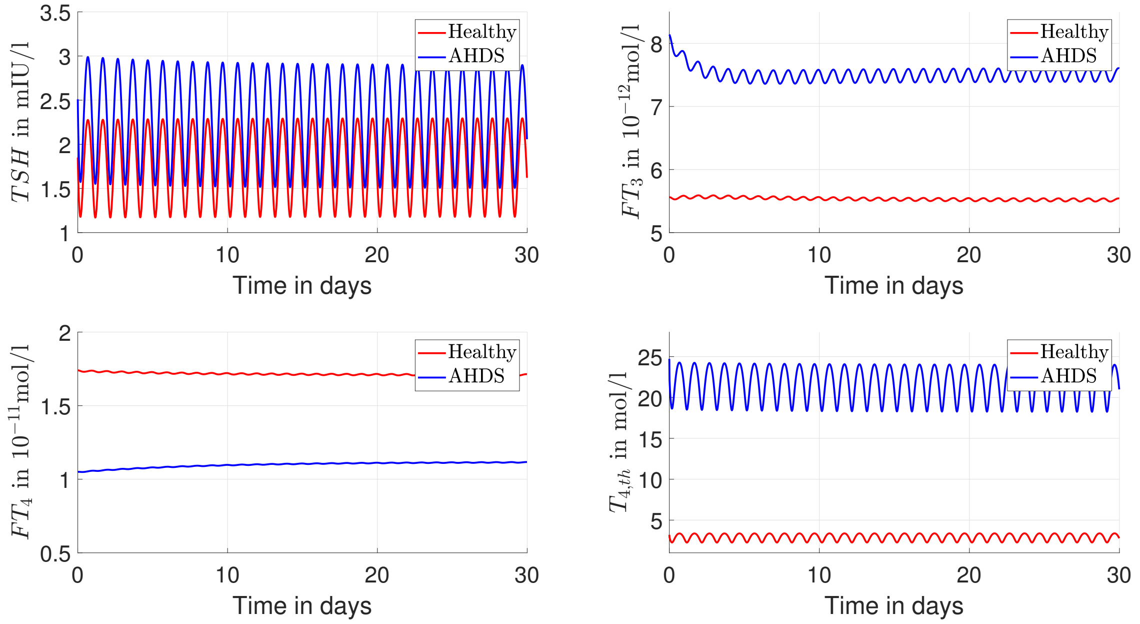

Figure 3. Results of the dynamic simulations of the linear modeling of the membrane transporter. Again, most of the numerical parameter values are based on (1, 3). The remaining parameters are estimated through a constrained parameter optimization approach and shown in Table S3.

S6 MCT8-MEDIATED T_3 TRANSPORT

As mentioned in the main part, we here consider additionally membrane transporters for T_3. To this end, we proceed similarly as for the membrane transporters for T_4. We introduce a new state called $T_{3,th}$ which

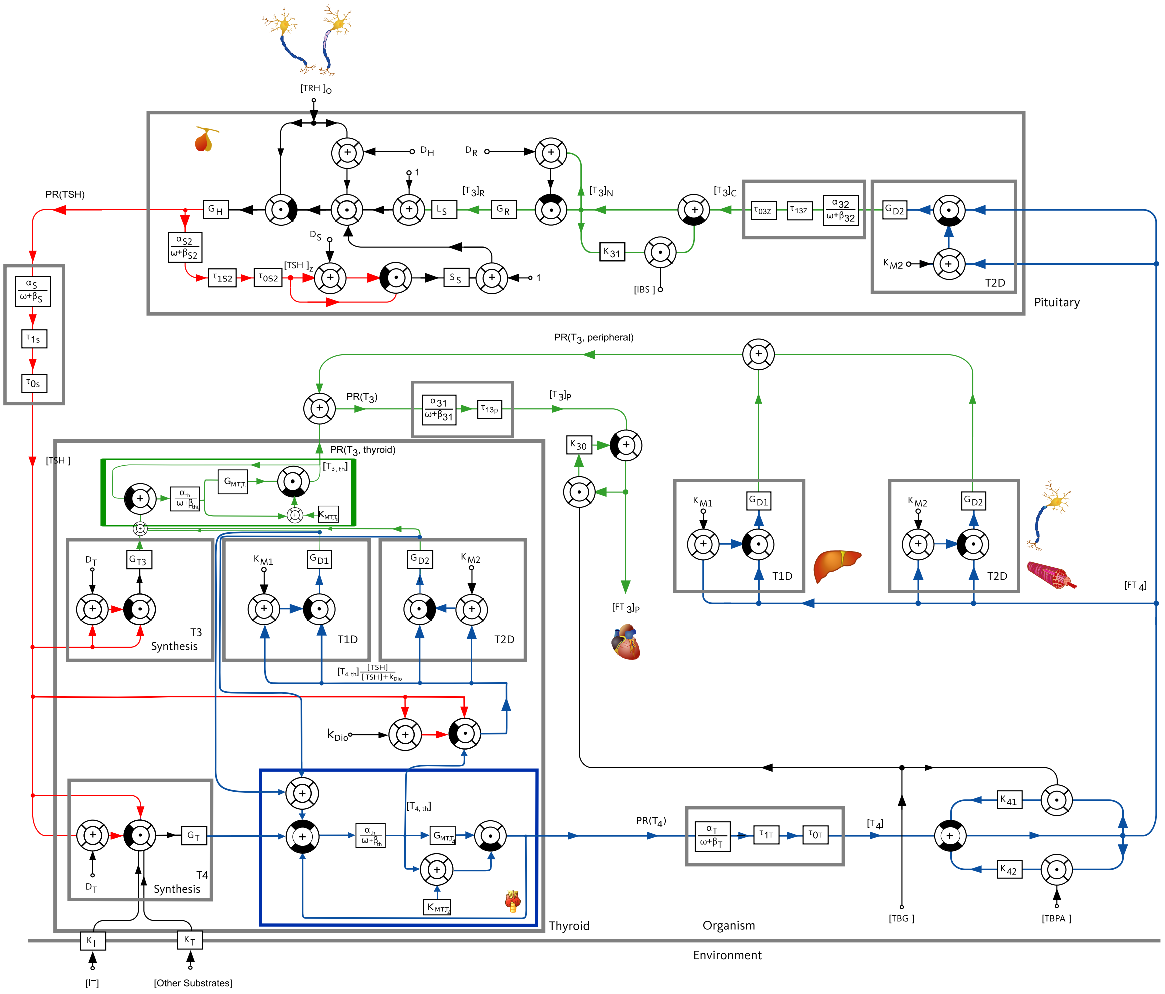

Figure 4. Block Diagram of the pituitary-thyroid feedback loop if membrane transporters for T_3 (illustrated by means of the green frame) and for T_4 (illustrated by means of the blue frame) are considered.

represents the T_3 concentration in thyroid cells. Its associated differential equation is

$$\frac{dT_{3,th}}{dt}(t) = \alpha_{th}\left(G_{D1}\frac{T_{4,th}(t)\frac{TSH(t)}{TSH(t)+k_{Dio}}}{T_{4,th}(t)\frac{TSH(t)}{TSH(t)+k_{Dio}} + K_{M1}} + G_{D2}\frac{T_{4,th}(t)\frac{TSH(t)}{TSH(t)+k_{Dio}}}{T_{4,th}(t)\frac{TSH(t)}{TSH(t)+k_{Dio}} + K_{M2}} + G_{T3}\frac{TSH(t)}{D_T + TSH(t)} - G_{MT,T_3}\frac{T_{3,th}(t)}{T_{3,th}(t) + K_{MT,T_3}}\right) - \beta_{th,T_3}T_{3,th}(t) \tag{S44}$$

The production of T_3 in thyroid cells depends on (i) the conversion of T_4 into T_3 in thyroid cells and (ii) and a direct T_3 synthesis based on the TSH concentration. The dilution factor α_{th} is the same as in the differential equation of $T_{4,th}$ (since we assume that the intrathyroidal T_3 has the same volume of distribution as the intrathyroidal T_4). The choice of β_{th,T_3} is more complicated. From (12), we can deduce that the intrathyroidal half-life of T_4 is approximately 44 hours, which is much shorter than the plasma half-life of T_4 corresponding approximately to 7 days. Assuming that the intrathyroidal half-life of T_3 is reduced in the same way (compared to the plasma half life of T_3), its half-life would correspond to approximately 6 hours

and the corresponding clearance rate to $\beta_{th,T_3} = 3.06 \cdot 10^{-5}\mathrm{s}^{-1}$. Once again, slightly different numerical parameter values will lead to the same qualitative behavior[3].

Next, the differential equation of the peripheral T_3 concentration (called T_{3p}) must be adapted. We now consider positively the part of $T_{3,th}$ which is transported out of the thyroid cells and the peripheral conversion of T_4 into T_3, yielding

$$\frac{dT_{3p}}{dt}(t) = \alpha_{31}\left(G_{D1}\frac{FT_4(t)}{FT_4(t)+K_{M1}} + G_{D2}\frac{FT_4(t)}{FT_4(t)+K_{M2}} + G_{MT,T_3}\frac{T_{3,th}(t-\tau_{03P})}{T_{3,th}(t-\tau_{03P})+K_{MT,T_3}}\right) - \beta_{31}T_{3p}(t) \tag{S45}$$

containing the new parameters G_{MT,T_3} and K_{MT,T_3}. Once again, the value of K_{MT,T_3} has already been determined experimentally in (13) and its value is applied here (although this is a simplification since different mutations will lead to different sensitivities). The block diagram in Figure 4 illustrates the incorporation of membrane transporters for T_3 (compare the block with a green frame) and T_4 (compare the block with a blue frame).

The parameter G_{MT,T_3} describing the maximum activity of the T_3 transport must be estimated for healthy individuals. We estimate this parameter by following the same concept, as described in Section S3 (using cost function (S33)) with the only difference that we additionally identify the parameter G_{MT,T_3}. Subsequent simulations for one healthy individual subject are shown in Figure 5. In this figure, one can see that the modeled hormone concentrations still fit to the measured hormone concentrations. Additionally, one can still observe the circadian rhythm of FT_3. Hence, our model remains a valid approximation of the pituitary-thyroid feedback when considering $MCT8$-mediated T_3 transport.

Even though the hormone concentrations for healthy individuals can be accurately represented considering membrane transporters for T_3, the parameters become structurally non-identifiable. In other words, substantially different (of several orders of magnitude) parameter configurations lead to the same hormone concentrations. This difference to the estimation results when only membrane transporters for T_4 are considered is due to the additional parameter that we need to estimate, i.e., $G_{MT,T3}$, while using the same real hormone concentrations measurements. In other words, we want to estimate an additional parameter without further data or information.

Therefore, we do not consider membrane transporters for T_3 in the main part, although this approach is a simplification since it has been shown that the MCT8 transports T_3 in humans, compare (13). Nevertheless, this is a meaningful approach, since the T_3 export is most likely not harmed in $MCT8$-deficiency (14). This intriguing observation could be explained by the existence of further membrane transporters as the MCT10 (which transports T_3 and occurs in the thyroid gland (15)) or the recently discovered SLC17A4 (which also transports T_3 (16)).

S7 STABILITY ANALYSIS

As mentioned in the main part of this paper, we performed a local stability analysis. To this end, we introduce Lyapunov's indirect method. Then, we document and discuss the results from a physiological point of view.

[3] For example, if we assumed that the intrathyroidal T_3 half-life is the same than the plasma T_3 half-life, then we would still obtain the same qualitative results as presented in Figure 5.

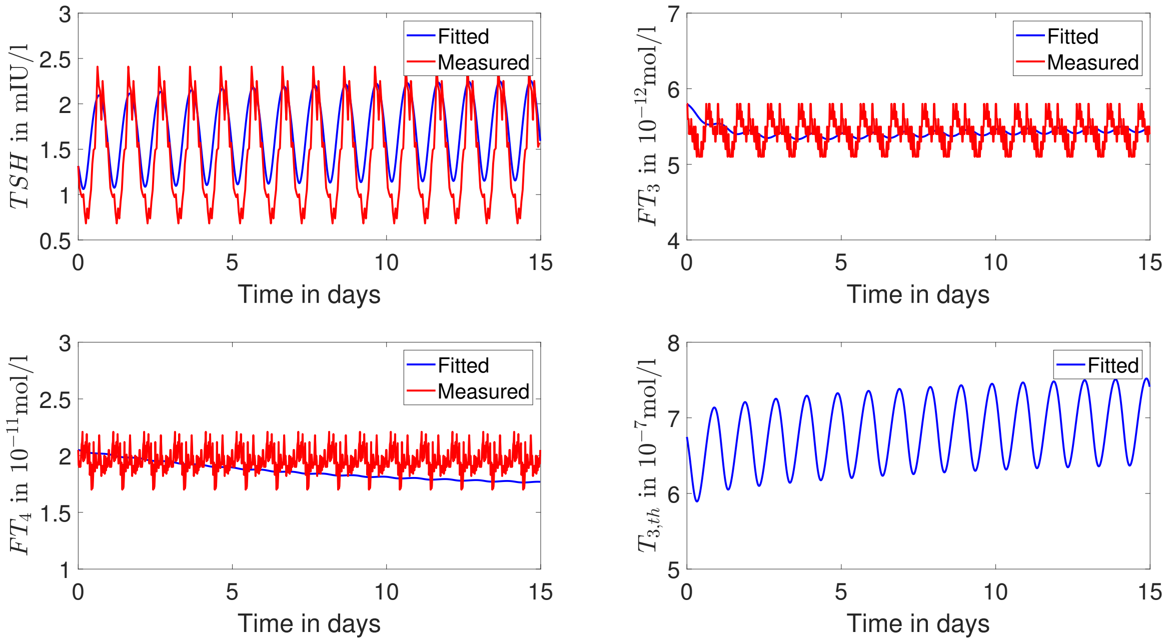

Figure 5. Simulation results for a model which considers explicitly the T_3 content in thyroid cells and the membrane transporters for T_3.

Lyapunov's Indirect Method

Lyapunov's indirect method can be formulated as follows (17): let $x = 0$ be an equilibrium point/hormone concentration for the nonlinear system

$$\dot{\mathbf{x}} = \mathbf{f}(\mathbf{x}) \tag{S46}$$

where $\mathbf{f} : D \mapsto \mathbb{R}^n$ is continuously differentiable and D is a neighborhood of the origin. Let

$$\mathbf{A} = \left.\frac{\partial \mathbf{f}(\mathbf{x})}{\partial \mathbf{x}}\right|_{\mathbf{x}=0}, \tag{S47}$$

then

1. the origin is locally exponentially stable if Re $\lambda_i < 0$ for all eigenvalues of $\mathbf{A}$.
2. The origin is unstable if Re $\lambda_i > 0$ for one or more of the eigenvalues of $\mathbf{A}$.

As Lyapunov's indirect method is only a criterion for local exponential stability, no statement is possible regarding the region of attraction of the equilibrium point. The region of attraction of an equilibrium point is the set of all initial states (here: hormone concentrations), for which the solution of the nonlinear system (S47) asymptotically converges to the equilibrium point.

Results Lyapunov's Indirect Method

The stability analysis concerns the equilibrium hormone concentrations documented in Tables 1 and 2 of the main part, which were obtained using a constant concentration of TRH. The application of Lyapunov's indirect method leads to the results illustrated in Table S5. One can see that the eigenvalues are negative for

Table S5. Results of the application of Lyapunov's indirect method.

	M.-M. Modeling of Membrane Transporters		Linear Modeling of Membrane Transporters	
Eigenvalue	Healthy Individuals	AHDS Patients	Healthy Individuals	AHDS Patients
λ_1	$-8.00 \cdot 10^{-6}$	$-8.00 \cdot 10^{-6}$	$-8.00 \cdot 10^{-6}$	$-8.00 \cdot 10^{-6}$
λ_2	$-2.46 \cdot 10^{2}$	$-2.50 \cdot 10^{2}$	$-2.46 \cdot 10^{2}$	$-2.50 \cdot 10^{2}$
λ_3	$-8.30 \cdot 10^{-4}$	$-8.30 \cdot 10^{-4}$	$-8.30 \cdot 10^{-4}$	$-8.30 \cdot 10^{-4}$
λ_4	$-1.46 \cdot 10^{-6}$	$-1.34 \cdot 10^{-6}$	$-1.43 \cdot 10^{-6}$	$-1.33 \cdot 10^{-6}$
λ_5	$-2.30 \cdot 10^{-4}$	$-2.30 \cdot 10^{-4}$	$-2.30 \cdot 10^{-4}$	$-2.30 \cdot 10^{-4}$
λ_6	-119.35	-18.58	-119.32	-18.50

Annotations: the abbreviation M.M. stands for Michaelis-Menten.

all cases. Consequently, the computed equilibrium hormone concentrations are all locally exponentially stable.

Discussion Stability Analysis

After having documented these (rather technical) results, we can focus on their medical interpretation.

Local asymptotic stability means that if the hormone concentrations have values different from their equilibrium hormone concentrations, which are still in the region of attraction, the hormone concentrations will converge to their equilibrium hormone concentrations. Unfortunately, with the proposed method it is impossible to state how large the region of attraction is. Hence, it is impossible to quantify the maximal deviation of the hormone concentrations for which they converge to their equilibrium hormone concentrations.

This result holds true for healthy individuals and for AHDS patients. This is particularly interesting since AHDS patients have a highly perturbed pituitary-thyroid feedback loop. From a systems theoretic point of view, their equilibrium points are still locally asymptotically stable.

In the future, it would be highly valuable to determine the mentioned region of attraction. A possible method that goes along with a quantification of the region of attraction is Lyapunov's direct method (17). However, its application is highly challenging if the differential equations are complex as it is the case here.

The aforementioned results hold for a constant TRH concentration. Since the real TRH concentration in humans follows a pulsatile course, one might ask how this time-varying TRH concentration impacts the stability results. To this end, we note the following. As discussed above, the application of Lyapunov's indirect method shows that the equilibrium hormone concentrations (with constant TRH input) are locally exponentially stable. This implies that the (nonlinear) system (S1) - (S9) comprising the pituitary-thyroid feedback loop is locally input-to-state stable (ISS) (which follows, e.g., by applying Lemma 4.6 of (17)). This property (ISS), which has become one of the standard methods to quantify a certain notion of robustness of nonlinear systems, implies that small/bounded (but potentially time-varying) inputs (or deviations from a constant equilibrium input) result in small/bounded states (or deviations from the equilibrium state). In other words, small (potentially time-varying) deviations of TRH around a constant equilibrium value (e.g., a pulsatile course) result in small deviations of the hormone concentrations.

S8 DERIVATION OF THE STEADY STATE EQUATION OF TSH

Here the step-by-step solution to derive the steady-state equation of TSH in dependence of $T_{4,th}$ is presented for the Michaelis-Menten modeling of the membrane transporters.

The steady-state equation of T_4 is

$$T_4 = \frac{\alpha_T}{\beta_T} G_{MT} \frac{T_{4,th}}{K_{MT} + T_{4,th}}, \tag{S48}$$

which follows from setting (S2) to zero and solving the equation for T_4. This steady-state equation can be plugged into the relationship of FT_4 (S8), thus leading to

$$FT_4 = \frac{\alpha_T}{\beta_T} G_{MT} \frac{T_{4,th}}{K_{MT} + T_{4,th}} \frac{1}{1 + K_{41} TBG + K_{42} TBPA}. \tag{S49}$$

With $b_1 = 1/(1 + K_{41}TBG + K_{42}TBPA)$, equation (S49) can be written as

$$FT_4 = \frac{\alpha_T}{\beta_T} G_{MT} \frac{T_{4,th}}{K_{MT} + T_{4,th}} b_1. \tag{S50}$$

This can be used for the steady state equation of T_{3c}, which can again be computed by setting (S4) to zero and solving for T_{3c}. When the relation of FT_4 (S50) is plugged into this steady-state equation, one gets

$$T_{3c} = a_3 \frac{\frac{\alpha_T}{\beta_T} G_{MT} \frac{T_{4,th}}{K_{MT}+T_{4,th}} b_1}{\frac{\alpha_T}{\beta_T} G_{MT} \frac{T_{4,th}}{K_{MT}+T_{4,th}} b_1 + K_{M2}} \tag{S51}$$

with $a_3 = \alpha_{32} G_{D2}/\beta_{32}$. By defining $p_1 = \alpha_T b_1/\beta_T$, equation (S51) simplifies to

$$T_{3c} = a_3 \frac{p_1 G_{MT} \frac{T_{4,th}}{K_{MT}+T_{4,th}}}{p_1 G_{MT} \frac{T_{4,th}}{K_{MT}+T_{4,th}} + K_{M2}}. \tag{S52}$$

Next, the expression of T_{3N} (S9) is used. With $b_3 = 1/(1 + K_{31}IBS)$ and expression (S52), T_{3N} is

$$T_{3N} = b_3 T_{3c} \tag{S53}$$

$$= b_3 a_3 \frac{p_1 G_{MT} \frac{T_{4,th}}{K_{MT}+T_{4,th}}}{p_1 G_{MT} \frac{T_{4,th}}{K_{MT}+T_{4,th}} + K_{M2}}. \tag{S54}$$

The term $G_R T_{3N}/(T_{3N} + D_R)$ is needed for the steady-state computation of TSH. With the definition of $p_2 = a_3 b_3 p_1$ and $p_3 = p_2 + D_R p_1$, it becomes

$$G_R \frac{T_{3N}}{T_{3N}+D_R} = G_R \frac{b_3 a_3 \frac{p_1 G_{MT} \frac{T_{4,th}}{K_{MT}+T_{4,th}}}{p_1 G_{MT} \frac{T_{4,th}}{K_{MT}+T_{4,th}} + K_{M2}}}{b_3 a_3 \frac{p_1 G_{MT} \frac{T_{4,th}}{K_{MT}+T_{4,th}}}{p_1 G_{MT} \frac{T_{4,th}}{K_{MT}+T_{4,th}} + K_{M2}} + D_R} \tag{S55}$$

$$= G_R \frac{b_3 a_3 p_1 G_{MT} \frac{T_{4,th}}{K_{MT}+T_{4,th}}}{b_3 a_3 p_1 G_{MT} \frac{T_{4,th}}{K_{MT}+T_{4,th}} + D_R (p_1 G_{MT} \frac{T_{4,th}}{K_{MT}+T_{4,th}} + K_{M2})} \tag{S56}$$

$$= G_R \frac{p_2 G_{MT} \frac{T_{4,th}}{K_{MT}+T_{4,th}}}{p_3 G_{MT} \frac{T_{4,th}}{K_{MT}+T_{4,th}} + D_R K_{M2}}. \tag{S57}$$

Now the steady-state value of TSH can be computed, which again follows from setting (S5) to zero and solving for TSH. With the following relationships

$$p_4 = G_H \frac{\alpha_S}{\beta_S} \frac{TRH_0}{TRH_0 + D_H} \tag{S58}$$

$$g_7 = \frac{\alpha_{S2} \beta_S}{\alpha_S \beta_{S2}} \tag{S59}$$

$$TSH_z = \frac{\alpha_{S2} \beta_S}{\alpha_S \beta_{S2}} TSH = g_7 TSH \tag{S60}$$

the steady-state equation of TSH is

$$TSH = p_4 \frac{g_7 TSH + D_S}{g_7 TSH (1 + S_S) + D_S} \frac{1}{1 + L_S G_R \frac{T_{3N}}{T_{3N}+D_R}}. \tag{S61}$$

With the expression (S57) and the definition of the constants

$$p_5 = g_7 (1 + S_S) \tag{S62}$$

$$p_6 = p_3 + p_2 L_S G_R \tag{S63}$$

$$p_7 = D_R K_{M2} \tag{S64}$$

$$p_8 = p_4 g_7 p_3 \tag{S65}$$

$$p_9 = p_4 g_7 D_R K_{M2} \tag{S66}$$

$$p_{10} = p_4 D_S p_3 \tag{S67}$$

$$p_{11} = p_4 D_S D_R K_{M2}, \tag{S68}$$

one gets the following equation

$$TSH^2(G_{MT}\frac{T_{4,th}}{K_{MT}+T_{4,th}}p_5p_6+p_5p_7)+TSH\big(G_{MT}\frac{T_{4,th}}{K_{MT}+T_{4,th}}(D_Sp_6-p_8)+ D_Sp_7-p_9\big)-G_{MT}\frac{T_{4,th}}{K_{MT}+T_{4,th}}p_{10}-p_{11}=0. \tag{S69}$$

By defining some final constants

$$q_1=p_5p_6 \tag{S70}$$

$$q_2=p_5p_7 \tag{S71}$$

$$q_3=D_Sp_6-p_8 \tag{S72}$$

$$q_4=D_Sp_7, \tag{S73}$$

one obtains the more compact steady-state equation (S38)

$$TSH^2(G_{MT}\frac{T_{4,th}}{K_{MT}+T_{4,th}}q_1+q_2)+TSH(G_{MT}\frac{T_{4,th}}{K_{MT}+T_{4,th}}q_3+q_4-p_9) - G_{MT}\frac{T_{4,th}}{K_{MT}+T_{4,th}}p_{10}-p_{11}=0. \tag{S74}$$

S9 NUMERICAL PARAMETER VALUES

Symbol	Description	Value	Origin
TBG	Concentration of thyroxine-binding globulin	$300\,\mathrm{nmol/L}$	(18)/ well known reference value
$TBPA$	Concentration of Transthyretin	$4.5\ \mu\mathrm{mol/L}$	(18)/ well known reference value
IBS	Concentration of intra-cellular T_3-binding substrate	$8\ \mu\mathrm{mol/L}$	Estimated form TBG-concentration, corrected for intra-cellular T_3-accumulation (according to values from (19))
TRH_0	TRH-concentration in hypophyseal portal system	$6.9\ \mathrm{nmol/s}$	(20)
G_H	Secretory capacity of the pituitary	$817\ \mathrm{mIU/s}$	Calculated according to (21) and (22)
D_H	Damping constant (EC_{50}) of TRH at the pituitary	$47\ \mathrm{nmol/s}$	(23)
α_S	Dilution factor for peripheral TSH	$0.4\,\mathrm{L}^{-1}$	Reciprocal value of the volume of distribution of 2.5 L (1)
β_S	Clearance exponent for peripheral TSH	$2.3 \cdot 10^{-4}\ \mathrm{s}^{-1}$	Calculated from plasma half-life of 50 min (24, 25)
L_S	Brake constant of long feedback	$1.68\ \mathrm{L}/\mu\mathrm{mol}$	Calculated from clinical data of hyperthyroid patients (1)
G_T	Secretory capacity of thyroid gland	Compare main part	Fitted to real measurements
D_T	Damping constant (EC_{50}) at the thyroid gland	$2.75\,\mathrm{mIU/L}$	(26)
α_T	Dilution factor for T_4	$0.1\,\mathrm{L}^{-1}$	Reciprocal value of the volume of distribution (27)
β_T	Clearance exponent for T_4	$1.1 \cdot 10^{-6}\mathrm{s}^{-1}$	Calculated from plasma half-life of 7 days (27, 28)
K_{M1}	Dissociation constant of 5'-deiodinase type I	$500\,\mathrm{nmol/L}$	(27)
α_{31}	Dilution factor for peripheral T_3	$2.6 \cdot 10^{-2}\ \mathrm{l}^{-1}$	Reciprocal value of volume of distribution (27)
β_{31}	Clearance exponent for peripheral T_3	$8 \cdot 10^{-6}\ \mathrm{s}^{-1}$	Calculated from plasma half-life of 24 h (1)
G_{D2}	Maximum activity of 5'deiodinase type II	$4.3\,\mathrm{fmol/s}$	Calculated form pituitary T_3-concentration (29)
K_{M2}	Dissociation constant of 5'-deiodinase type II	$1\,\mathrm{nmol/L}$	(30)
α_{32}	Dilution factor for central T_3	$1.3 \cdot 10^{5}\ \mathrm{L}^{-1}$	Calculated from volume of distribution 7.6 µl (1)

Symbol	Description	Value	Origin
β_{32}	Clearance exponent for central T_3	$8.3 \cdot 10^{-4}\,\mathrm{s}^{-1}$	Calculated from intra-cellular half-life of 15 min (31, 32)
α_{S2}	Dilution factor for pituitary TSH	$2.6 \cdot 10^{5}\,\mathrm{l}^{-1}$	Calculated from volume of distribution (1) of $3.8\mu l$
β_{S2}	Clearance exponent for pituitary TSH	$140\,\mathrm{s}^{-1}$	Estimated, corresponding to half-life of $5\,\mathrm{ms}$ (1)
D_R	Damping constant for central T_3	$100\,\mathrm{pmol/L}$	(33)
G_R	Maximum gain of $TR\beta$-receptors	$1\,\mathrm{mol/s}$	Value unknown, normalized to 1 (magnitude of feedback is determined by L_S) (1)
S_S	Brake constant of ultrashort feedback	$100\,\mathrm{L/mIU}$	Determined according to values from (34)
D_S	Damping constant for TSH inside the pituitary	$50\,\mathrm{mIU/L}$	Determined according to values from (34)
K_{30}	Dissociation constant T_3-TBG	$2 \cdot 10^{9}\mathrm{L/mol}$	(25)
K_{31}	Dissociation constant T_3-IBS	$2 \cdot 10^{9}\,\mathrm{L/mol}$	Value unknown, adapted to extra-cellular dissociation constant (1)
K_{41}	Dissociation constant T_4-TBG	$2 \cdot 10^{10}\,\mathrm{L/mol}$	(25)
K_{42}	Dissociation constant T_4-$TBPA$	$2 \cdot 10^{8}\mathrm{L/mol}$	(25)
τ_{0S}	Peripheral delay for TSH	120 s	Derived from circulation time (1)
τ_{0S2}	Pituitary delay for TSH	3240 s	Derived from period of TSH-pulses (data from (35))
τ_{0T}	Delay for T_4	300 s	Estimated according to circulation and diffusion times (1)
τ_{03Z}	Delay for pituitary T_3	3600 s	Derived from (36)
τ_{03P}	Delay for peripheral T_3	300 s	Derived from (37)
α_{th}	Dilution factor for $T_{4,th}$	$250\,\mathrm{L}^{-1}$	Based on an assumed volume of distribution of $4\,\mathrm{ml}$
β_{th}	Clearance Exponent for $T_{4,th}$	$4.4 \cdot 10^{-6}\mathrm{s}^{-1}$	Calculated from intrathyroidal half-life of 44 h (12)
k_{Dio}	Stimulation constant of thyroidal D1 and D2	$1\,\mathrm{mIU/L}$	(3)
K_{MT}	Michaelis-Menten constant membrane transporter	$4.7 \cdot 10^{-6}\,\mathrm{mol/L}$	(38)
G_{D1}	Maximum activity of 5'deiodinase type I	Compare main part	Fitted to real measurements
G_{T3}	Maximum activity of direct T_3 synthesis	Compare main part	Fitted to real measurements
G_{MT}	Maximum activity of the membrane transporters	Compare main part	Fitted to real measurements
k_l	Linear approximation constant membrane transporters	Compare main part	Fitted to real measurements

REFERENCES

1 .Dietrich JW. *Der Hypophysen-Schilddrüsen-Regelkreis: Entwicklung und klinische Anwendung eines nichtlinearen Modells*. Ph.D. thesis (2001). doi:10.13140/RG.2.1.4845.9368.

2 .Dietrich JW, Tesche A, Pickardt CR, Mitzdorf U. Thyrotrpic feedback control: evidence for an additional ultrashort feedback loop from fractal analysis. *Cybernetics and Systems* **35** (2004) 315–331. doi:10.1080/01969720490443354.

3 .Berberich J, Dietrich JW, Hoermann R, Müller MA. Mathematical modeling of the pituitary-thyroid feedback loop: Role of a TSH-T3-shunt and sensitivity analysis. *Frontiers in endocrinology* **9** (2018) 91–91. doi:10.3389/fendo.2018.00091.

4 .Russell W, Harrison RF, Smith N, Darzy K, Shalet S, Weetman AP, et al. Free triiodothyronine has a distinct circadian rhythm that is delayed but parallels thyrotropin levels. *The Journal of clinical endocrinology and metabolism* **93** (2008) 2300–6.

5 .Pilo A, Iervasi G, Vitek F, Ferdeghini M, Cazzuola F, Bianchi R. Thyroidal and peripheral production of 3,5,3'-triiodothyronine in humans by multicompartmental analysis. *The American journal of physiology* **258** (1990) E715–26.

6 .Bianco AC, Salvatore D, Gereben B, Berry MJ, Larsen PR. Biochemistry, cellular and molecular biology, and physiological roles of the iodothyronine selenodeiodinases. *Endocrine reviews* **23** (2002) 38–89.

7 .Maranduba CMdC, Friesema EC, Kok F, Kester MH, Jansen J, Sertie AL, et al. Decreased cellular uptake and metabolism in allan-herndon-dudley syndrome (ahds) due to a novel mutation in the mct8 thyroid hormone transporter. *Journal of Medical Genetics* **43** (2006) 457–460.

8 .Chen X, Liu L, Zeng C. A novel variant in slc16a2 associated with typical allan-herndon-dudley syndrome: a case report. *BMC pediatrics* **22** (2022) 1–5.

9 .Anık A, Kersseboom S, Demir K, Çatlı G, Yiş U, Böber E, et al. Psychomotor retardation caused by a defective thyroid hormone transporter: report of two families with different mct8 mutations. *Hormone Research in Paediatrics* **82** (2014) 261–271.

10 .Rego T, Lado CG, Rodríguez PC, Santos FS, Angueira FB, Castro-Feijóo L, et al. Severe neurological abnormalities in a young boy with impaired thyroid hormone sensitivity due to a novel mutation in the mct8 gene. *Hormones* **16** (2017) 194–199.

11 .Azzolini S, Nosadini M, Balzarin M, Sartori S, Suppiej A, Mardari R, et al. Delayed myelination is not a constant feature of allan–herndon–dudley syndrome: report of a new case and review of the literature. *Brain and Development* **36** (2014) 716–720.

12 .Di Cosmo C, Liao XH, Dumitrescu AM, Philp NJ, Weiss RE, Refetoff S. Mice deficient in MCT8 reveal a mechanism regulating thyroid hormone secretion. *The Journal of clinical investigation* **120** (2010) 3377–88.

13 .Kinne A, Kleinau G, Hoefig CS, Grüters A, Köhrle J, Krause G, et al. Essential molecular determinants for thyroid hormone transport and first structural implications for monocarboxylate transporter 8. *Journal of Biological Chemistry* **285** (2010) 28054–28063.

14 .Trajkovic-Arsic M, Müller J, Darras VM, Groba C, Lee S, Weih D, et al. Impact of monocarboxylate transporter-8 deficiency on the hypothalamus-pituitary-thyroid axis in mice. *Endocrinology* **151** (2010) 5053–5062. doi:10.1210/en.2010-0593.

15 .Friesema EC, Jansen J, Jachtenberg Jw, Visser WE, Kester MH, Visser TJ. Effective cellular uptake and efflux of thyroid hormone by human monocarboxylate transporter 10. *Molecular endocrinology* **22** (2008) 1357–1369.

16 .Groeneweg S, van Geest FS, Chen Z, Farina S, van Heerebeek RE, Meima ME, et al. Functional characterization of the novel and specific thyroid hormone transporter slc17a4. *Thyroid* **32** (2022) 326–335.

17 .Khalil HK. *Nonlinear systems* (Upper Saddle River, NJ: Prentice Hall), 3 edn. (2002).

18 .Neubert D. Schilddrüsenhormon. *Endokrinologie II* **19** (1977) 65–212.

19 .M T Hays JT MR Broome. A multicompartmental model for iodide, thyroxine, and triiodothyronine metabolism in normal and spontaneously hyperthyroid cats. *Endocrinology* **122** (1988) 2444–2461. doi:10.1210/endo-122-6-2444.

20 .Rondell JMM, De Greff WJ, Van Der Schoot P, Karels B, Klootwijk W, Visser TJ. Effect of thyroid status and paraventricular area lesions on the release of thyrotropin-releasing hormone and catecholamines into hypophysial portal blood. *Endocrinology* **123** (1988) 523–527. doi:10.1210/endo-123-1-523.

21 .D'angelo S, Paul D, Wall N, Lombardi D. Pituitary thyrotropin (TSH) rebound phenomenon and kinetics of secretion in the goitrous rat: differential effects of thyroxine on synthesis and release of TSH. *Endocrinology* **99** (1976) 935–943.

22 .Okuno A, Taguchi T, Nakayama K, Takimoto M. Kinetic analysis of plasma TSH dynamics after TRH stimulation. *Hormone and Metabolic Research* **11** (1979) 293–295.

23 .Le Dafniet M, Brandi AM, Kujas M, Chanson P, Peillon F. Thyrotropin-releasing hormone (TRH) binding sites and thyrotropin response to TRH are regulated by thyroid hormones in human thyrotropic adenomas. *European journal of endocrinology* **130** (1994) 559–564.

24 .Odell WD, Utiger RD, Wilber JF, Condliffe PG. Estimation of the secretion rate of thyrotropin in man. *J Clin Invest* **46** (1967) 953–959. doi:10.1172/JCI105601.

25 .Li G, Liu B, Liu Y. A dynamical model of the pulsatile secretion of the hypothalamo-pituitary-thyroid axis. *Bio Systems* **35** (1995) 83–92.

26 .Dumont JE, Vassart G. Thyroid regulation. *Endocrinology DeGroot, WB Saunders* (1995).

27 .Greenspan F. Basic & clinical endocrinology, chapter the thyroid gland. *Stamford, CT, Appleton & Lange* (1997) 192–262.

28 .Grussendorf M. *Metabolismus der Schilddrüsenhormone 1* (Stuttgart, New York: Thieme) (1988).

29 .Doorn JV, Roelfsema F, Heide DVD. Concentrations of thyroxine and 3, 5, 3-triiodothyronine at 34 different sites in euthyroid rats as determined by an isotopic equilibrium technique. *Endocrinology* **117** (1985) 1201–1208.

30 .Visser TJ, Kaplan MM, Leonard JL, Larsen PR. Evidence for two pathways of iodothyronine 5-deiodination in rat pituitary that differ in kinetics, propylthiouracil sensitivity, and response to hypothyroidism. *J Clin Invest* **71** (1983) 992–1002. doi:10.1172/JCI110854.

31 .Oppenheimer JH, Bernstein G, Hasen J. Estimation of rapidly exchangeable cellular thyroxine from the plasma disappearance curves of simultaneously administered thyroxine-131i and albumin-125i. *J Clin Invest* **46** (1967) 762–777. doi:10.1172/JCI105577.

32 .Benvenga S, Robbins J. Thyroid hormone efflux from monolayer cultures of human fibroblasts and hepatocytes. Effect of lipoproteins and other thyroxine transport proteins. *Endocrinology* **139** (1998) 4311–4318. doi:10.1210/endo.139.10.6231.

33 .Lazar MA, Chin WW, et al. Nuclear thyroid hormone receptors. *The Journal of clinical investigation* **86** (1990) 1777–1782.

34 .Kakita T, Laborde N, Odell WD. Autoregulatory control of thyrotropin in rabbits. *Endocrinology* **114** (1984) 2301–2305. doi:10.1210/endo-114-6-2301.

35 .Greenspan SL, Klibansk A, Schqenfeld D, Ridgway E. Pulsatile secretion of thyrotropin in man. *J Clin Endocrinol Metab* **63** (1986) 661–668. doi:10.1210/jcem-63-3-661.

36 .Larsen PR. Thyroid-pituitary interaction: feedback regulation of thyrotropin secretion by thyroid hormones. *New England Journal of Medicine* **306** (1982) 23–32.

37 .Westgren U, Burger A, Ingemansson S, et al. Preoperative studies on thyroid activity: Secretion of T4, T3 and 'reverse t3' rT3 in normal man. *Thyroid Research. Excerpta Medica* (1976) 226–228.

38 .Friesema ECH, Ganguly S, Abdalla A, Manning Fox JE, Halestrap AP, Visser TJ. Identification of monocarboxylate transporter 8 as a specific thyroid hormone transporter. *The Journal of biological chemistry* **278** (2003) 40128–35.
